# *Brucella* infection induces chromatin restructuring in host cells to activate immune responses

**DOI:** 10.3389/fimmu.2025.1574006

**Published:** 2025-06-05

**Authors:** Dejian Xie, Heling Xu, Changwei Su, Jingjing Lu, Wenlong Shen, Ping Li, Bingyu Ye, Jiabao Hou, Junwei Deng, Yan Zhang, Shanhu Li, Zhihu Zhao

**Affiliations:** ^1^ Laboratory of Advanced Biotechnology, Beijing Institute of Biotechnology, Beijing, China; ^2^ College of Life Sciences, Henan Normal University, Henan, China

**Keywords:** *Brucella*, chromatin restructuring, interferon-stimulated genes, 3D genome, host-pathogen interactions

## Abstract

**Background:**

*Brucella* spp., facultative intracellular pathogens that cause brucellosis, drive pathogenesis by invading host cells and establishing intracellular persistence. While their molecular mechanisms are well-characterized, how *Brucella* induces chromatin restructuring in host cells remains poorly understood, representing a critical gap in host-pathogen interaction research.

**Methods:**

Using an established *in vitro* infection model of *Brucella*-infected RAW264.7 murine macrophages, we integrated Hi-C, ATAC-seq, and RNA-seq to generate multi-omics datasets. Multidimensional comparative genomics approaches were employed to systematically map infection-induced changes in host chromatin architecture and functional genomic organization.

**Results:**

Our findings unveiled substantial alterations in the host chromatin architecture, characterized by a reduction in B-B compartment regions interactions, an increase in A-B compartment interactions, and diminished long-range chromatin contacts. Crucially, *Brucella* reshaped chromatin compartmentalization, activating interferon-stimulated genes (ISGs) in regions transitioning from compartment B to A. Enhanced sub-TADs interactions within ISG clusters further facilitated their coordinated expression. Additionally, infection remodeled chromatin loop structures, strengthening interactions linked to immune-related gene activation.

**Conclusion:**

These results demonstrate that host cells undergo substantial chromatin remodeling during acute *Brucella* infection as a defense mechanism against pathogen invasion. Our findings provide critical insights into host-pathogen interactions and suggest potential epigenetic targets for managing brucellosis.

## Introduction

Brucellosis is an infectious bacterial disease caused by *Brucella* species, characterized by systemic invasion in humans and animals. It is one of the most common zoonotic diseases in the world. Research estimates that there are approximately 2.1 million new human cases annually worldwide, with 43.2% of the global population potentially at risk (1). Notably, about 10-30% of acute brucellosis patients eventually progress to a chronic phase of the disease (2).

*Brucella* primarily infects macrophages within the host. Following phagocytosis, the majority of bacteria are rapidly targeted for lysosomal destruction. However, a subset evades this initial macrophage defense, establishing replication within a specialized vacuolar niche that circumvents the endocytic pathway (3). The pathogen orchestrates intracellular survival by forming *Brucella*-containing vacuoles (BCVs), which prevent lysosomal fusion and subsequent degradation (3). Host cells activate interferon signaling pathways and upregulate interferon-stimulated genes (ISGs) to restrict bacterial replication (4). Concurrently, the host cells secrete large amounts of cytokines to recruit immune cells to clear the infected cells. Nevertheless, the molecular mechanisms driving rapid immune activation during *Brucella* infection remain incompletely characterized.

Gene expression is regulated via chromatin-based mechanisms within the nuclei of eukaryotic cells. Chromatin is intricately organized within the cell nucleus, forming hierarchical structures including chromatin territories, A/B compartments, topologically associating domains (TADs), and chromatin loops (5, 6). Chromatin territories arise from the spatial folding of individual chromatin fibers into distinct nuclear regions. A/B compartments, which correlate with euchromatin and heterochromatin states, exhibit spatial clustering of similar compartment types, creating distinct interaction patterns. CTCF, a key architectural protein, plays a critical role in demarcating the boundaries of TADs and chromatin loops (7). Cohesin complexes dynamically engage with CTCF-bound sites, stabilizing chromatin architecture through their loop-extruding activity (8). The 3D organization of chromatin modulates biological processes such as transcription (9), DNA replication (10), cell division (11), meiosis (11, 12) and DNA damage repair (13–15), which are crucial for cell differentiation, animal development and onset of diseases (16). Pathogen infections can restructure host chromatin to counteract host immunity and facilitate their own replication or long-term persistence. In turn, host cells can modify their chromatin structure to activate the expression of immune genes and eliminate pathogens. Such as SARS-CoV-2, can restructure the host chromatin conformation and inhibit the expression of IFN genes by reducing loop interactions and contacts within TADs (17). In contrast, *Mycobacterium tuberculosis* can activate the NF-κB signaling pathway to restructure the host cell chromatin and promote the expression of immune-related genes (18).

While previous studies have demonstrated that infection by various pathogenic microorganisms can remodel host chromatin architecture and modulate gene expression, it remains unclear whether *Brucella* infection alters host chromatin conformation and, if so, whether such changes coordinately activate immune-related genes. To address these questions, we propose an integrated multi-omics approach: Hi-C to map genome-wide chromatin interactions, ATAC-seq to profile chromatin accessibility dynamics, and RNA-seq to identify differentially expressed genes during *Brucella* infection. This integrated approach enabled systematic investigation of Brucella-induced host chromatin reorganization and comprehensive delineation of infection-associated epigenetic reprogramming. Our findings reveal that *Brucella* infection triggers pronounced chromatin remodeling in host cells, establishing an epigenetic framework essential for orchestrating anti-bacterial immune responses.

## Materials and methods

### Brucella cultivation and quantification

The *Brucella* strain utilized in this study was *Brucella melitensis* 16M (sheep strain 1116). *Brucella* cultures were grown in *Brucella* broth to mid-log phase. Bacterial density was measured at OD600 and adjusted to 1×10^9^ CFU/mL. The inoculum was calculated based on the predetermined multiplicity of infection (MOI) of 100. To prepare the bacterial suspension, the culture was centrifuged at 12,000 × g for 1 minute to pellet the bacteria. The supernatant was discarded, and the bacterial pellet was resuspended in phosphate-buffered saline (PBS). This step was repeated by centrifuging at 12,000×g for 1 minute and discarding the supernatant. Finally, the bacterial pellet was resuspended in Dulbecco’s Modified Eagle Medium (DMEM) culture medium.

### Cell culture and bacterial infection

*Brucella melitensis* infection of RAW264.7 macrophages is a well-established and widely validated infection model (19–21). Therefore, we selected RAW264.7 cells as the experimental subject in this study. The mouse macrophage cell line RAW264.7 was cultured in DMEM medium supplemented with 10% fetal bovine serum (FBS) for 2 days. The cells were then trypsinized, re-counted, and seeded at a density of 1×10^7 cells per dish for overnight culture. After the cells adhered to the dish, the culture medium was discarded, and the cells were washed three times with PBS. The cells were then incubated in DMEM medium without FBS. Mock control and *Brucella* infection groups were established. For the *Brucella* infection group, the bacterial suspension was added to the culture dish, gently mixed, and incubated in the cell culture incubator for 2 hours. The mock group received an equal volume of DMEM medium. After the infection period, the bacterial-containing medium was removed, and the cells were washed 3–5 times with PBS. The cells were then incubated in DMEM medium containing 50 µg/ml gentamicin and 10% FBS for 2 hours in the incubator. The medium was subsequently replaced with complete medium, and the cells were cultured for an additional 48 hours.

### Hi-C3.0 experiment

We have modified the standard Hi-C3.0 experimental protocol (22). Specifically, we replaced DSG with Ethylene Glycol-bis(Thermo Scientific, Cat# 21556) for the cross-linking reaction. Additionally, after de-crosslinking, we used magnetic beads to extract DNA instead of the phenol: chloroform and isoamyl alcohol mixture.

In brief, cultured cells were harvested, the culture medium was removed, and the cells were washed with PBS. A 10 ml solution of 1% formaldehyde was added for cross-linking at room temperature for 10 minutes. The cross-linking was quenched with glycine to a final concentration of 128 mM, mixed thoroughly, and then placed on ice for 15 minutes. The cross-linked cells were subjected to a second cross-linking with 3 mM EGS for 40 minutes, followed by quenching with glycine to a final concentration of 400 mM. The cross-linked samples were lysed with lysis buffer (10 mmol/L Tris-HCl (pH 8.0), 10 mmol/L NaCl, 0.2% NP40, 1% PIC). The cells were then homogenized using a cell grinder to obtain nuclei. The nuclei were treated with 0.1% SDS at 65°C for 10 minutes, followed by neutralization with 0.1% Triton X-100. The sample was centrifuged at 1000×g for 5 minutes at 4°C to remove the supernatant. The genome was digested with 200U *DdeI* (New England Biolabs, Cat# R0175L) and 200U *DpnII* restriction enzymes (New England Biolabs, Cat# R0543M) for 16 hours. The digestion was terminated by incubating at 65°C for 20 minutes. The DNA ends were blunted with 10U Klenow (New England Biolabs, Cat# M0210L) at 23°C for 4 hours. Biotin labeling was performed by supplementing the reaction system with 8 mM Bio-14-dATP(Jean Bioscience, Cat# NU-835-BIO14-L), enabling efficient incorporation of biotin tags for subsequent detection and isolation procedures. The enzyme was then heat-inactivated at 75°C for 20 minutes. The nuclei were collected and ligated with 100U T4 DNA ligase overnight at 16°C. The sample was treated with 20 µL proteinase K at 65°C for 4 hours to degrade the proteins. DNA was purified using the Qiagen DNeasy Mini Kit. The biotinylated ends were removed using T4 DNA polymerase, and the DNA was recovered using 1× VAHTS DNA Clean beads (Vazyme, Cat# N411-03). The DNA was then end-repaired and blunted using the VAHTS Universal DNA library Prep Kit for Illumina V3 (Vazyme, Cat# ND607). Biotinylated chimeric fragments were enriched using streptavidin C1 beads (Invtrogen, Cat# 2844953). The enriched fragments were amplified by PCR (95°C for 3 minutes, followed by 8 cycles of 98°C for 20 seconds, 60°C for 15 seconds, and 72°C for 30 seconds, with a final extension at 72°C for 5 minutes and hold at 4°C). The amplified products were purified using 1× VAHTS DNA Clean Beads and sequenced on the Illumina Novaseq 6000 platform. Four biological replicates were performed for each experiment.

### ATAC-seq library preparation

The ATAC library was constructed using the Hyperactive ATAC-Seq Library Prep Kit for Illumina (Vazyme, Cat# TD711) according to the manufacturer’s protocol. Cells were dissociated using trypsin and counted with an automated cell counter. A total of 100,000 cells were collected for subsequent processing. The cells were centrifuged at 2,300 rpm for 5 minutes at 4°C, after which the supernatant was carefully removed. The cell pellet was then washed twice by resuspension in Tween buffer.

Cell membranes were lysed using lysis buffer (0.1% NP-40, 0.1% Tween-20, 0.01% digitonin). Following lysis, nuclei were collected by low-speed centrifugation at 2,300 rpm for 10 minutes at 4°C. The nuclear pellet was resuspended in transposase reaction mixture containing 0.5 μL 10% Tween-20, 0.5 μL 1% digitonin, 10 μL 5× TTBL buffer, and 4 μL TTE Mix V50, followed by tagmentation at 37°C for 30 minutes.

The fragmented DNA was purified using ATAC DNA Extract Beads and amplified by 10 cycles of PCR. Final libraries were sequenced on the Illumina NextSeq 6000 platform. Five biological replicates were performed for each experiment to ensure reproducibility and statistical robustness.

### RNA-seq library preparation

The culture medium was aspirated from the cell dish, and the cells were gently washed with phosphate-buffered saline (PBS) to remove residual medium. Total RNA was extracted using TRIzol reagent (Invitrogen, Cat# 10057841) according to the manufacturer’s instructions. RNA integrity and concentration were assessed using a Bioanalyzer. RNA-seq libraries were prepared using the VAHTS^®^ Universal RNA-Seq Library Prep Kit for Illumina (Vazyme, Cat# NR605-01) following the manufacturer’s instructions. The library was sequenced on the Illumina NovaSeq 6000 platform. Four biological replicates were performed for each experiment to ensure data reliability and reproducibility.

### Hi-C data analysis

The raw sequencing data were subjected to quality control and preprocessing using the fastp (v 0.23.4) software (23) to remove adapter sequences and low-quality reads. High-quality reads were subsequently aligned to the mouse reference genome (mm10) using the bwa-mem2 (v2.2.1) alignment tool. The aligned reads were further processed and analyzed using the cooler (v0.9.2) software suite to construct interaction matrices. Pair-reads were binned into cool files at multiple resolutions, including 1 kb, 2 kb, 5 kb, 10 kb, 20 kb, 40 kb, 100 kb, 200 kb, and 1 Mb. These interaction matrices were normalized to correct for biases and facilitate downstream analysis.

### Construction of *Brucella* genome interaction map

We generated the chromatin interaction map of *Brucella melitensis* through the following pipeline: Raw sequencing reads were first aligned to the *Brucella melitensis* reference genome using bwa-mem2 (v2.2.1). The aligned data were then processed and normalized with cooler to construct genome-wide interaction matrices at 100-bp and 500-bp resolutions. Finally, interaction heatmaps were visualized through matplotlib package.

### ATAC-seq data analysis

Raw sequencing data were subjected to quality control and adapter trimming using the fastp (v0.23.4) software. High-quality reads were aligned to the mouse reference genome (GRCm38/mm10) or *Brucella melitensis* 16M genome(GCA_000250815) using the bowtie2 (v2.3.5.1) alignment tool. Peaks were called using the macs3 (v3.0.1) software (24), and differential peaks between the Brucella-infected and mock-treated groups were analyzed using the Diffbind (v3.8.1) package. Enrichment analysis of differential peaks was performed using the deeptools (v3.5.1) suite (25).

### RNA-Seq data analysis

Raw sequencing data were processed using the fastp (v0.23.4) software to remove adapters and low-quality reads. Filtered reads were aligned to the mm10 reference genome using HISAT2 (v2.2.1) (26), a rapid and efficient alignment tool. Transcripts were assembled using StringTie2 (v2.2.3) (27), which provides accurate quantification and assembly of RNA-Seq data. Transcript abundance was quantified using the RSEM (v1.2.28) software (28). Differential expression analysis was performed using DESeq2 (v1.38.0) (29), which provides robust normalization and statistical testing for differential expression. Gene ontology (GO) annotation and Kyoto Encyclopedia of Genes and Genomes (KEGG) pathway analysis of differentially expressed genes were conducted using the clusterProfiler (v4.6.2) package (30).

### A/B compartment identification

Normalized 10-kilobase (kb) matrices were generated using the cooler balance module. The expected matrix was obtained using the cooltools expected-cis module. Compartment signals within chromosomes (cis) were calculated through eigenvalue decomposition using the cooltools eigs-cis function. A/B compartments were determined based on gene density within gene loci, with A compartments typically associated with transcriptionally active regions and B compartments with transcriptionally repressive regions.

### TAD and subTAD identification

TADs and sub-TADs were identified using the insulation function of the cooltools (v0.5.1) software (31) on 10-kb and 2-kb matrices. The domain structure and boundaries of sub-TADs were further analyzed using the coolpup.py tool. This approach allowed for the precise delineation of chromatin interaction domains and their hierarchical organization within the genome.

### Loop identification

Chromatin loops were identified using the mustache (v1.1.0) software (32) with the following parameters: –pThreshold 0.1, –sparsityThreshold 0.88 and –octaves 2. Differential loops were identified using the diff_mustache.py script from the mustache package. This parameter configuration was empirically selected to balance detection sensitivity (FDR < 5%) and specificity through systematic benchmarking with positive control datasets.

### Genomic locus visualization

Genomic loci were visualized using matplotlib for Python and R. These tools were employed to generate high-resolution heatmaps, interaction matrices, and other relevant visualizations to facilitate the interpretation of chromatin interaction data.

### Statistical analysis and reproducibility

No data were excluded during the analysis. Four to five biological replicates were constructed for all omics data to ensure robustness and reproducibility. Statistical analyses were executed with methodologically matched hypothesis tests (two-tailed Student’s t-test for parametric data; Mann-Whitney U test for non-parametric distributions), applying rigorous significance thresholds (p < 0.05 with Benjamini-Hochberg FDR correction).

## Results

### *Brucella* infection restructures host chromatin architecture

**Figure 1:**
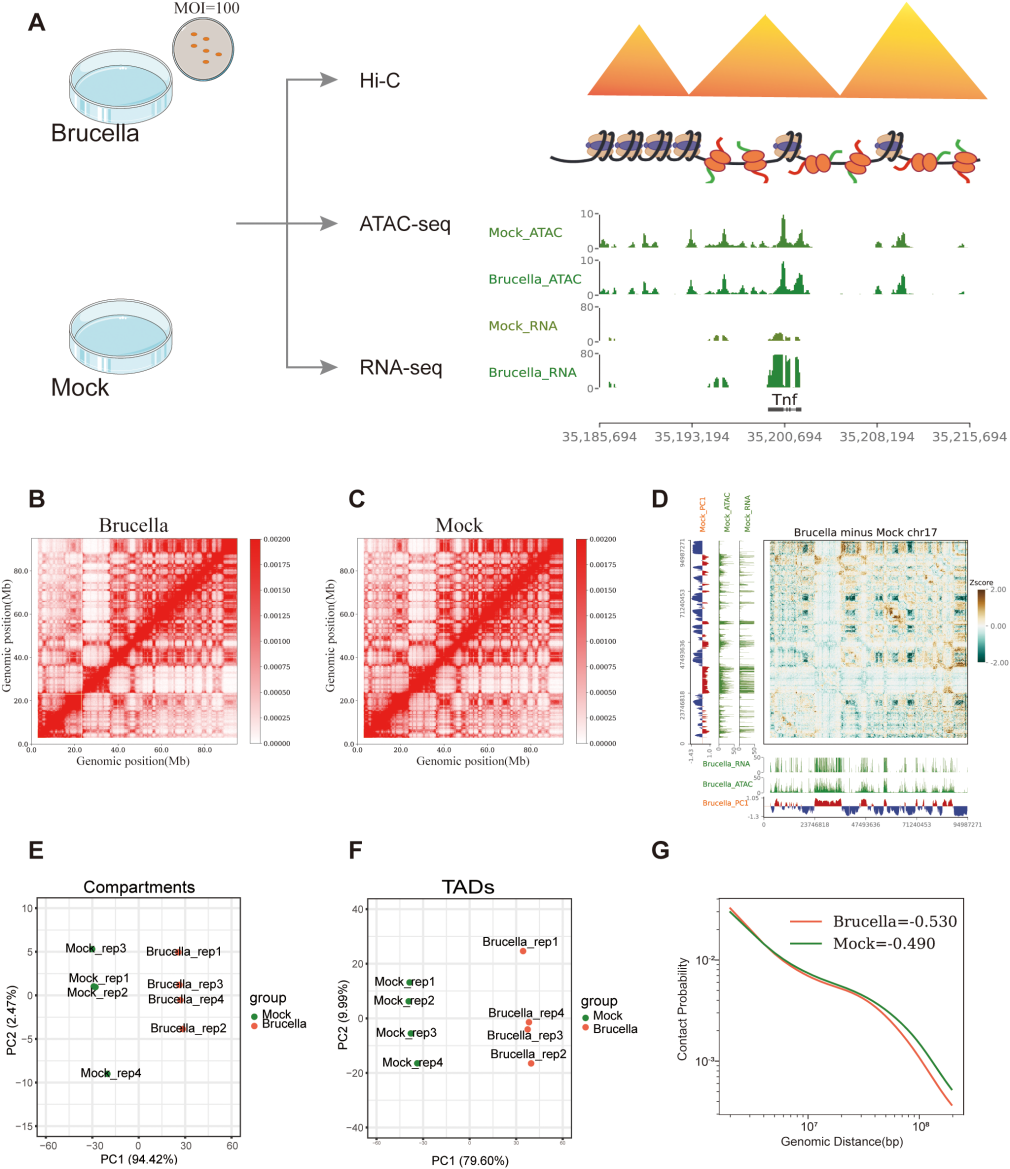
*Brucella* infection restructures the host cell genome. **(A)** Experimental design of the study. The figure outlines the overall experimental framework, including sample preparation, infection conditions, and analytical workflows for Hi-C, ATAC-Seq, and RNA-Seq analyses. **(B)** Interaction map of chromosome 17 in the *Brucella*-infected group, with a resolution of 200 kb. The heatmap represents the frequency of interactions between genomic regions, with warmer colors indicating higher interaction frequencies. **(C)** Interaction map of chromosome 17 in the mock-treated group, with a resolution of 200 kb. The heatmap represents the frequency of interactions between genomic regions, with warmer colors indicating higher interaction frequencies. **(D)** Differential interaction map of chromosome 17 between the *Brucella*-infected and mock-treated groups. The z-score difference map highlights regions with significant changes in interaction frequency. Yellow areas represent increased interactions in the infected group, while green areas represent decreased interactions in the infected group. **(E)** PCA of compartment PC1 values in samples from the *Brucella*-infected and mock-treated groups. The plot illustrates the separation of samples based on PC1 of compartmentalization, highlighting changes in chromatin compartment structure. **(F)** PCA of insulation scores in samples from the *Brucella*-infected and mock-treated groups. The plot illustrates the separation of samples based on the PC1 of insulation scores, highlighting changes in TAD boundary strength. **(G)** P(s) curve analysis. The figure shows the relationship between the Hi-C contact frequency (P) of intrachromosomal interactions sorted by genomic distance (s) for the Brucella-infected group (tomato) and the mock-treated group (green). The curve illustrates the decay of interaction frequency with increasing genomic distance, highlighting differences in chromatin interaction patterns between the two conditions.

To elucidate the epigenetic alterations and their potential biological implications in host cells following *Brucella* infection of immune cells, we conducted a 48-hour infection of the mouse macrophage cell line RAW264.7 with *Brucella*. Post-infection, the cells exhibited a marked reduction in proliferation and an increased formation of pseudopodia structures (**Supplementary Figures 1A, B**). We utilized Hi-C 3.0 and ATAC-seq to characterize the three-dimensional chromatin architecture and accessibility landscape, complemented by RNA-seq to profile transcriptomic alterations (**Figure 1A**).

For the Hi-C libraries, we constructed four independent libraries, each sequenced to approximately 250 Gb of data. Collectively, Total valid interactions reached 1.811 × 10^9^ (infected) and 1.627 × 10^9^ (mock) with cis/total ratios >85% (**Supplementary Table 1**), confirming the successful construction of the libraries. The biological reproducibility of the Hi-C libraries was validated by the corrected correlation scores determined by HiCRep software (35) (**Supplementary Figure 1C**).

Hi-C analysis uncovered reorganization of the host 3D chromatin architecture following *Brucella* infection. The differential interaction heatmap for chromosome 17 demonstrated a significant decrease in B-B homotypic interactions (**Figures 1B–D**). Conversely, the differential heatmap for chromosome 2 indicated a significant enhancement in interactions between certain A compartments (**Supplementary Figures 1E–G**). Further dimensionality reduction of the A/B Compartment PC1 values revealed that PC1 accounted for 94.42% of the variance between the infected and mock-treated groups (**Figure 1E**). Utilizing the insulation score of TADs for PCA dimensionality reduction, we observed that PC1 explained 79.60% of the variance (**Figure 1F**). These results demonstrate that *Brucella* infection induces genome-wide chromatin restructuring, significantly altering both compartmentalization patterns and TAD organization.

The scaling plot of chromatin contact density with increasing genomic distance indicated that, relative to the mock group, the *Brucella*-infected group exhibited stronger short-distance chromatin contacts and marginally weaker interactions for far-separated regions. This suggests that *Brucella* infection facilitates local compaction of the host chromatin (**Figure 1G**). By separately analyzing chromatin interactions and interaction decay with distance within A/B compartments, we found that both A and B compartments in the infected group had stronger short-distance chromatin contacts. Additionally, the B compartment in the infected group displayed a more rapid decay of interactions (Chromatin interaction decay exponent, *Brucella*: Mock = -0.7615: -0.6208) (**Supplementary Figure 1D**). Finally, we examined intragenomic interactions within *Brucella* and found that sequencing data aligned to the *Brucella* genome in infected samples could be used to construct global interaction maps (**Supplementary Figure 1H**). Notably, chromatin accessibility signals were enriched at *Brucella* gene body loci (**Supplementary Figure 1I**), demonstrating effective bacterial infection. Furthermore, *Brucella* persisted intracellularly even after 48 hours post-infection.

In summary, our results demonstrate that *Brucella* infection can extensively remodel the host chromatin structure across multiple dimensions.

### Brucella infection promotes activation of host immune genes

**Figure 2:**
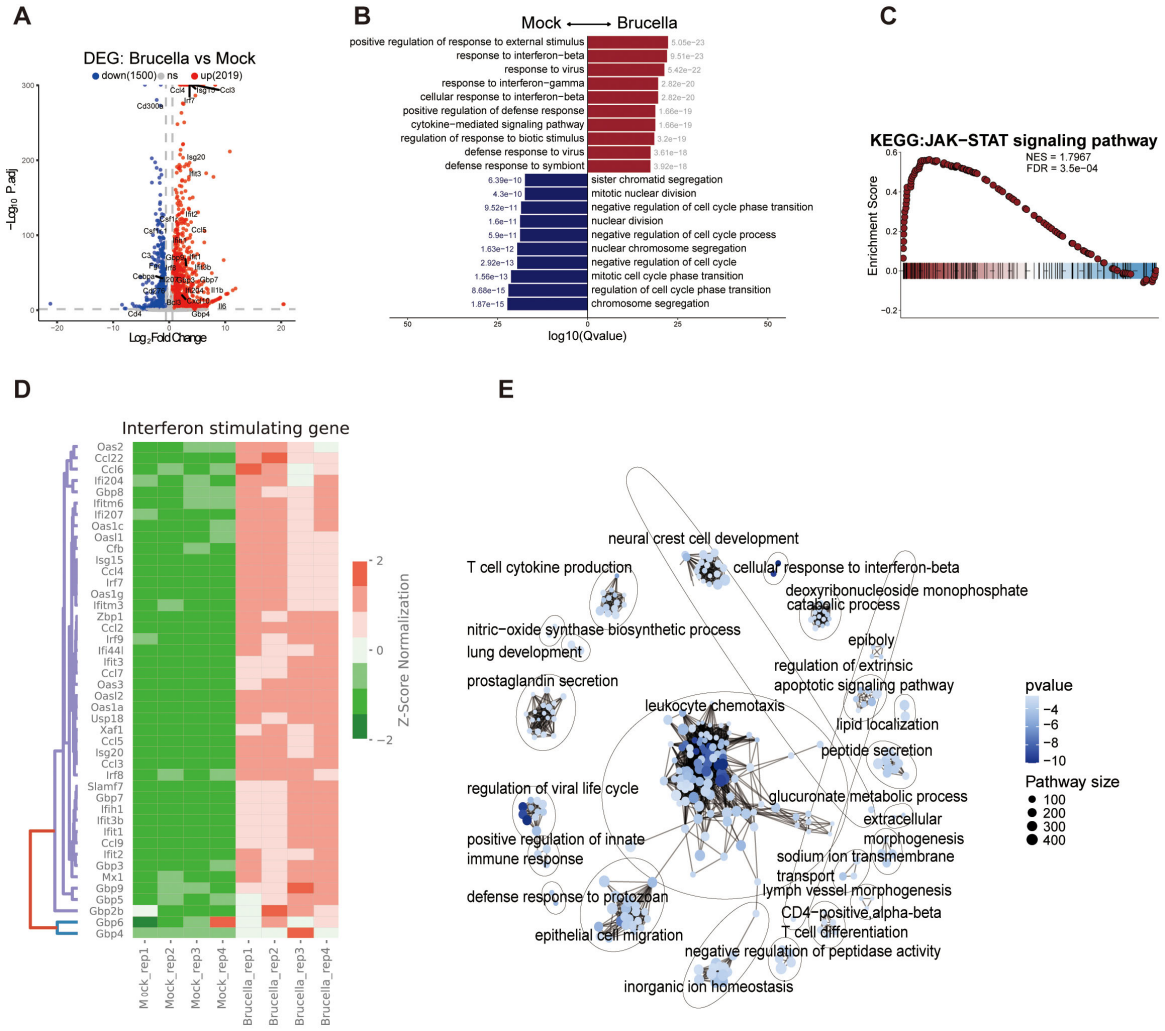
*Brucella* infection promotes the activation of immune genes. **(A)** Volcano plot depicting differentially expressed genes (DEGs) in response to Brucella infection. The plot illustrates the statistical significance (−log10 *P*-value) versus the fold change of gene expression, highlighting genes that are significantly upregulated and downregulated. **(B)** Bar plot displays the top 10 significantly enriched biological processes (GO terms) from DEGs between *Brucella*-infected and mock-infected groups. Blue bars represent *Brucella*-specific DEG enrichments, while red bars indicate mock-specific DEG enrichments. **(C)** Gene Set Enrichment Analysis (GSEA) enrichment plot showing significant overrepresentation of genes associated with the JAK-STAT signaling pathway. The plot highlights the enrichment score and the position of genes within the ranked list. **(D)** Heatmap of ISGs expression between *Brucella*-infected and mock-infected groups. The color bar represents the expression levels normalized by z-score, illustrating the relative expression changes across samples. **(E)** Simplified network diagram summarizing the GSEA results. The diagram illustrates key pathways and gene sets that are significantly enriched in response to *Brucella* infection, highlighting the interconnectedness of immune signaling pathways.

*Brucella* infection induces significant alterations in host cell phenotypes and chromatin conformation. To determine whether *Brucella* infection influences host gene expression, we conducted Spearman correlation analysis on gene expression data across samples, which revealed a clear distinction between *Brucella* infected and mock groups (**Supplementary Figure 2A**). Principal component analysis was employed to reduce the dimensionality of gene expression data from both groups, demonstrating that the PC1 accounted for 56.71% of the variance between samples, indicating that *Brucella* infection induces substantial changes in the transcriptional profile. Using DESeq2 to identify genes with expression level differences greater than 0.5-fold, we identified 2,019 upregulated genes and 1,500 downregulated genes (**Figure 2A**).

The GO analysis of differentially expressed genes showed that *Brucella* infection significantly activated genes in immune - related pathways like “response to interferon - beta” and “response to interferon - gamma”. Conversely, it suppressed genes in cell - cycle - regulation pathways such as “mitotic cell cycle phase transition” and “chromosome segregation” (**Figure 2B**). This matches the lower cell density in infected cultures than the control group after 48 hours (**Supplementary Figures 1A, B**), implying *Brucella* infection hinders host cell division. Also, the KEGG enrichment analysis of DEGs revealed that *Brucella* - infected samples had significant enrichment of inflammation and innate - immune - related pathways, including the TNF, cytokine - cytokine receptor interaction, and IL - 17 signaling pathways (**Supplementary Figure 2C**). Thus, *Brucella* infection strongly triggers the host innate immune response.

The JAK-STAT signaling pathway plays a critical role in mediating downstream interferon signaling activation. Gene Set Enrichment Analysis (GSEA) revealed significant enrichment of the JAK-STAT signaling pathway, as well as pathways related to “activation of innate immune response”, “cell chemotaxis”, “response to interferon-beta” and “response to interferon-gamma” (**Figure 2C**, **Supplementary Figures 2E–H**). Further analysis of key genes within the JAK-STAT signaling pathway demonstrated significant activation of *Stat* gene family members, including *Stat1*, *Stat2*, and *Stat3*. Notably, genes encoding negative regulators of the JAK-STAT pathway, such as *Socs1*, *Socs2*, and *Socs3* were significantly upregulated (**Supplementary Figure 2D**). These findings highlight the complex interplay between *Brucella* infection and host immune signaling pathways.

We further investigated the expression alterations of interferon-stimulated genes and observed significant upregulation of members of the IFIT family (*Ifit1*, *Ifit2*, *Ifit3*), GBP family (*Gbp2b, Gbp3, Gbp4, Gbp5, Gbp6, Gbp7, Gbp8, Gbp9*), and OAS gene family (*Oas1a, Oas1c, Oas1g, Oas2, Oas3*) (**Figure 2D**). Notably, cytokine family members *Ccl2, Ccl3, Ccl4, Ccl5, Ccl7, Ccl9*, and *Ccl22* were also significantly activated following infection. Analysis of chromatin accessibility at the gene loci of *Ccl3, Ccl4* and *Ccl5* revealed that *Brucella* infection promotes chromatin opening at these loci, thereby facilitating gene expression (**Supplementary Figures 2I–K**). In contrast, *Apoe* expression was downregulated post-infection, accompanied by decreased chromatin accessibility in its upstream promoter region (**Supplementary Figure 2L**). APOE, a basic protein rich in arginine, is one of the human apolipoproteins and plays important roles in lipoprotein synthesis, secretion, processing, and metabolism, as well as in blood lipid metabolism (36). *Apoe* has also been reported to inhibit inflammation caused by SARS-CoV-2 and HCV (36, 37). Specifically, we observed that genes related to lysosomes, *Lyz1* and *Lyz2* were suppressed following *Brucella* infection, with significant inhibition of chromatin accessibility at their loci (**Supplementary Figures 2M–N**). This may facilitate the intracellular survival of *Brucella* within macrophages.

To systematically analyze the pathways enriched by differentially expressed genes, we utilized the aPEAR software (38) to visualize the GSEA enrichment analysis results. The key nodes of the enriched pathways were centered on leukocyte chemotaxis (**Figure 2E**), indicating that *Brucella* infection promotes the migration of immune cells to the site of infection.

Collectively, these findings suggest that the interferon signaling pathway is a critical immune pathway in mouse macrophages for controlling *Brucella* infection.

### Chromatin compartmentalization dynamics and ISGs expression regulation in response to *Brucella* infection

**Figure 3:**
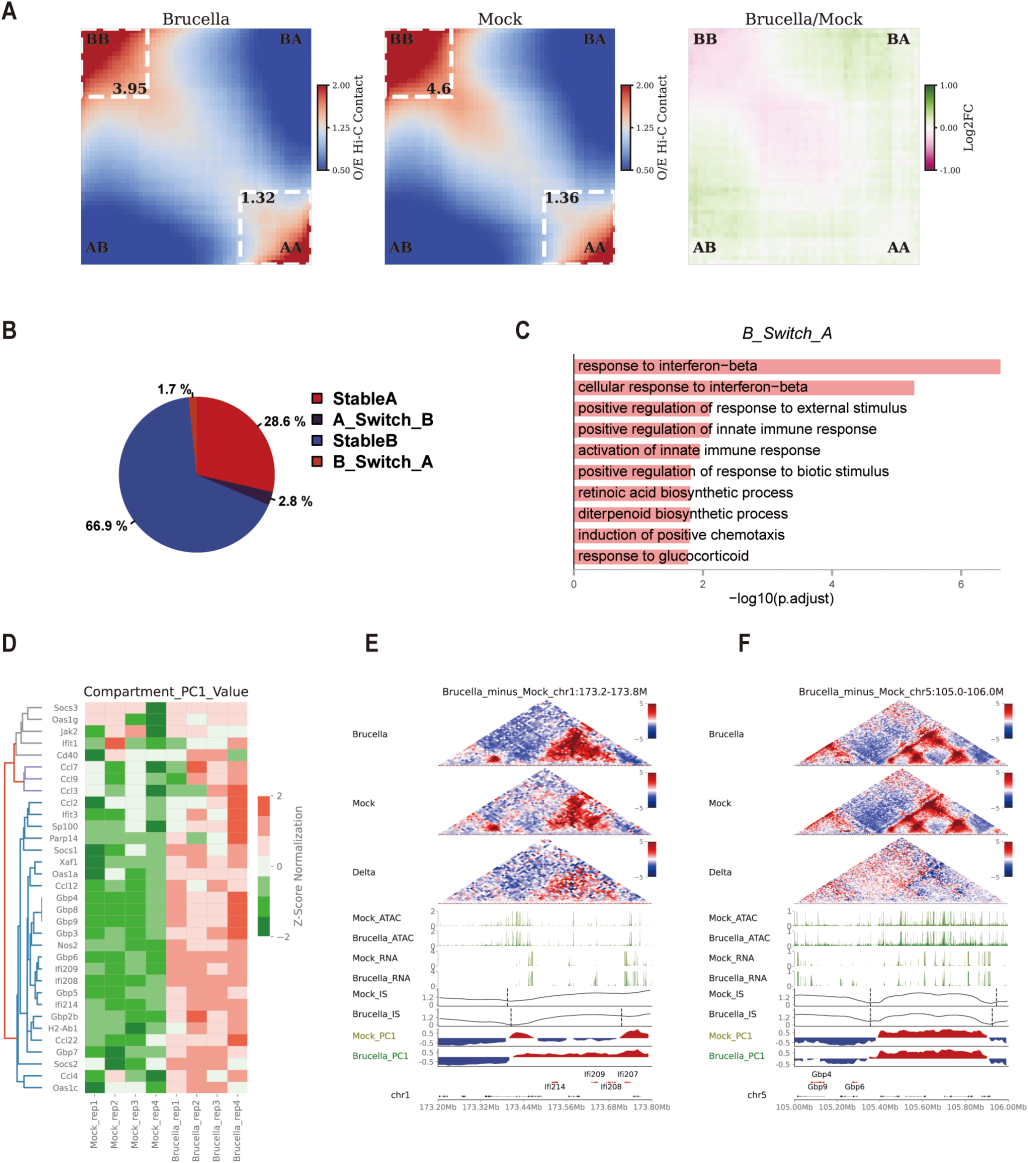
*Brucella* infection alters chromatin compartmentalization to promote activation of interferon-stimulated genes. **(A)** Saddle plot depicting chromatin compartmentalization between genomic regions, sorted by E1 score (the genome is divided into a total of 50 bins). A-A interactions are shown in the bottom right corner, while A-B interactions are located in the top right and bottom left corners. The right panel displays the log2(Brucella/Mock) difference score, highlighting changes in interaction frequencies between the two groups. **(B)** Pie chart illustrating the proportion of conserved and changed A/B compartments before and after infection. The chart provides an overview of the relative abundance of genomic regions that remain stable versus those that transition between compartments. **(C)** Gene Ontology (GO) annotations for upregulated genes in regions where B compartments transition to A compartments after infection. The analysis reveals enriched terms associated with immune response and signaling pathways, highlighting the functional relevance of these chromatin transitions. **(D)** Heatmap illustrating the changes in PC1 of ISG gene loci. The figure displays the Compartment PC1 values of specific gene promoter loci in the Mock and Brucella groups, normalized by z-score. The heatmap highlights shifts in chromatin compartmentalization associated with ISG activation. **(E)** Multi-omics data visualization for the chr1:173.2-173.8M region. In the interaction heatmap, the signals for both *Brucella*-infected and Mock samples were normalized by Z-score, while the Delta heatmap represents the differential interactions between *Brucella* and Mock groups (red: stronger interactions in *Brucella*; blue: stronger interactions in Mock). The ATAC track and RNA track show chromatin accessibility (ATAC-seq signal) and transcriptional activity (RNA-seq signal) in the region. The *Brucella*_IS and Mock_IS tracks display the insulation score (IS) signals of topologically associating domains in Brucella-infected and Mock control groups, respectively. The Brucella_PC1 and Mock_PC1 tracks represent the first principal component (PC1) signals in compartment analysis, with red indicating A compartments and blue denoting B compartments, respectively, while the Gene track annotates the genomic positions of genes. **(F)** Multi-omics data visualization for the chr5:105-106M region. In the interaction heatmap, the signals for both *Brucella*-infected and Mock samples were normalized by Z-score, while the Delta heatmap represents the differential interactions between *Brucella* and Mock groups (red: stronger interactions in *Brucella*; blue: stronger interactions in Mock). The ATAC track and RNA track show chromatin accessibility (ATAC-seq signal) and transcriptional activity (RNA-seq signal) in the region. The Brucella_IS and Mock_IS tracks display the insulation score (IS) signals of topologically associating domains in Brucella-infected and Mock control groups, respectively. The *Brucella*_PC1 and Mock_PC1 tracks represent the first principal component (PC1) signals in compartment analysis, with red indicating A compartments and blue denoting B compartments, respectively, while the Gene track annotates the genomic positions of genes.

Principal component analysis of Hi-C data enables the distinction of the genome into A/B compartments, with A compartments typically associated with transcriptionally active euchromatin and B compartments linked to transcriptionally repressive heterochromatin. Transitions between these compartments can significantly influence gene expression in the affected regions. Utilizing a 10-kb resolution matrix for compartment analysis, our saddle plot results demonstrated a marked reduction in interactions within B-B homotypic compartments, an increase in A-B heterotypic mixing interactions, and a slight rise in interactions within A-A compartments (**Figure 3A**). Further analysis of interactions within A and B compartments revealed a slight decrease in both (**Supplementary Figures 3A, B**), consistent with the decay index observations for A/B compartments (**Supplementary Figure 1D**). These findings suggest that *Brucella* infection induces significant reorganization of chromatin compartments.

Upon further investigation of A-B compartment transitions, we found that the majority of chromatin remained unchanged before and after infection (Stable A, 28.6%; Stable B, 66.9%). Only 1.7% of chromatin transitioned from A to B compartments, while 2.8% switched from B to A compartments (**Figure 3B**). Notably, GO annotation of upregulated genes in regions where B compartments switched to A compartments in the infected group revealed significant enrichment in interferon signaling pathways, such as “response to interferon-beta” (**Figure 3C**). This indicates that some ISGs were located in heterochromatin before *Brucella* infection and were activated following chromatin remodeling post-infection. Activated genes in stable A and stable B compartments were also enriched in pathways like “response to interferon-beta” or “cellular response to interferon-gamma” (**Supplementary Figures 3C, D**). Interestingly, we observed increased Compartment PC1 values at ISG loci following *Brucella* infection (**Figure 3D**), suggesting that enhanced PC1 values at these genomic loci correlate with gene activation.

Further analysis of the compartment vector in the promoter regions of ISGs showed a significant increase in PC1 values in the infected group, indicating a shift towards more euchromatic structures in these gene regions. This trend was evident in two ISG clusters (*Ifi214/Ifi207/Ifi208/Ifi209* and *Gbp3/Gbp5/Gbp7*) (**Supplementary Figures 3E, F**). Zooming in on the *Ifi214/Ifi207/Ifi208/Ifi209* gene locus, we observed enhanced interactions within this region post-infection (**Figure 3E**). Interestingly, another *Gbp* gene cluster (*Gbp4/Gbp6/Gbp9*) remained in the B compartment before and after infection, but their compartment values increased post-infection (**Figure 3D**). Zooming in on this gene region revealed its proximity to the A/B compartment boundary, with significant increased interactions with A compartments and enhanced PC1 values in the differential heatmap, suggesting that enhanced interactions between A/B compartments may promote gene expression in this region (**Figure 3F**).

In summary, *Brucella* infection induces significant remodeling of A/B compartment structures, thereby regulating the activation of ISG genes. This chromatin reorganization facilitates the transcriptional activation of ISGs, highlighting the critical role of chromatin dynamics in the host immune response to *Brucella* infection.

### Enhanced interactions within sub-TADs promote coordinated activation of ISGs cluster

**Figure 4:**
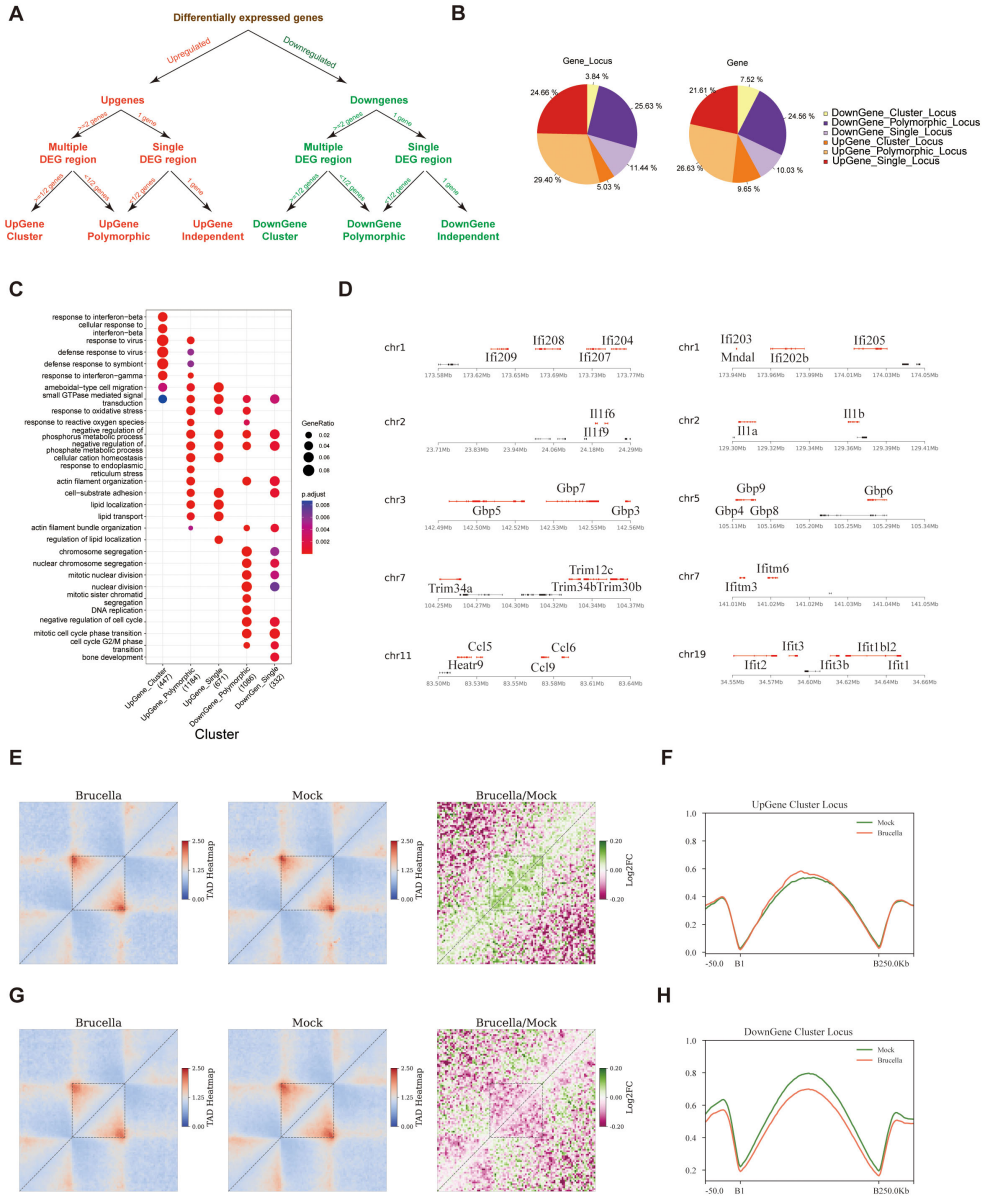
Changes in chromatin conformation promote coordinated activation of immune gene clusters. **(A)** Schematic representation of the strategy for identifying gene clusters, polymorphic gene loci, and independent gene loci. The approach involves systematic screening and categorization based on differential expression and chromatin interaction data. **(B)** Pie chart illustrating the distribution and proportion of gene loci identified in the study. The chart provides an overview of the relative abundance of different gene types within the dataset. **(C)** Dot plot displaying Gene Ontology annotations for six distinct categories of genes. The analysis reveals enrichment patterns, with the exception of Down_Cluster_Gene, which did not show significant enrichment under the specified screening conditions. **(D)** Visualization of selected gene clusters, with upregulated genes highlighted in red. The figure provides a spatial representation of gene loci and their expression status post-infection. **(E)** Analysis of intra-TAD interactions in the Upgene_Cluster_locus region using Aggregate Domain Analysis (ADA). The figure shows increased interaction frequency within this region after infection, with the dashed area indicating the sub-TADs. The left panel displays the stacking results in the Brucella group, the middle panel in the Mock group, and the right panel shows the log2 processing of the Brucella/Mock aggregate contact frequency. Green shading indicates regions with increased interaction frequency post-infection. **(F)** Post-infection, the average insulation index of the sub-TADs region containing UpGene_Cluster_Gene_locus is slightly upregulated. The figure illustrates the subtle changes in insulation scores, reflecting alterations in chromatin organization. **(G)** Analysis of intra-TAD interactions in the Downgene_Cluster_Gene_locus region using ADA. The figure shows changes in interaction frequency within this region after infection, with the dashed area indicating the sub-TADs. The left panel displays the stacking results in the Brucella group, the middle panel in the Mock group, and the right panel shows the log2 processing of the Brucella/Mock aggregate contact frequency. Red shading indicates regions with decreased interaction frequency post-infection. **(H)** Post-infection, the average insulation index of the sub-TADs region containing DownGene_Cluster_Gene_locus is significantly reduced. The figure highlights substantial changes in insulation scores, reflecting significant alterations in chromatin organization and potential regulatory impacts on gene expression.

To determine if *Brucella* infection triggers coordinated gene expression, we systematically analyzed the distribution of differentially expressed genes across the genome. We first calculated the structure of chromatin sub-TADs using a high-resolution matrix with a 2-kb resolution and then mapped upregulated and downregulated genes to these sub-TADs. Secondly, we classified the sub-TADs containing differentially expressed genes into UpGene loci and DownGene loci. A sub-TAD containing two or more differentially expressed genes (DEGs) with consistent expression changes (i.e., either upregulated or downregulated genes exceeding half of the total genes within the sub-TADs) was defined as a cluster gene locus. If a sub-TAD contained only one gene, and that gene was differentially expressed, it was defined as an independent gene locus. A sub-TAD with differentially expressed genes but where these genes did not exceed half of the total number of genes was defined as a polymorphic gene locus (**Figure 4A**).

Statistical results showed that DownGene Cluster Locus accounted for 3.84% of sub-TADs containing differentially expressed genes, representing 7.52% of all differentially expressed genes. UpGene Cluster Locus accounted for 5.03% of sub-TADs containing differentially expressed genes, representing 9.65% of all differentially expressed genes (**Figure 4B**). Interestingly, GO annotation analysis revealed that genes located in UpGene cluster locus were significantly enriched in pathways such as “response to interferon-beta” (**Figure 4C**). Further examination of the genomic locations of upregulated ISGs showed that these genes tend to cluster within the same sub-TADs (**Figure 4D**).

To elucidate the functional interplay between chromatin organization and transcriptional regulation, we performed a comprehensive analysis of sub-TADs encompassing genes with differential expression patterns. Intriguingly, highly expressed genes were preferentially located in sub-TADs with both significantly stronger intra-domain interaction intensities (**Supplementary Figure 4A**) and elevated insulation scores (**Supplementary Figure 4B**). These findings support a model wherein sub-TAD structural integrity facilitates local gene expression. Further analysis revealed that sub-TADs containing upregulated cluster gene locus showed increased internal interactions following *Brucella* infection (**Figure 4E**), with a slightly increase in insulation scores (**Figure 4F**) and a slight increase in ATAC signals (**Supplementary Figure 4G**). In contrast, sub-TADs containing downregulated gene clusters exhibited decreased internal interactions (**Figure 4G**), significantly lower insulation scores (**Figure 4H**), and a slight increase in ATAC signals (**Supplementary Figure 4H**). Similar patterns were observed in polymorphic gene locus and independent gene locus (**Supplementary Figures 4C–F**). Thus, we speculate that enhanced sub-TADs interactions promote enhancer-promoter interactions, thereby coordinately regulating the expression of related genes within the sub-TADs.

In summary, we found that *Brucella* infection enhances the internal interactions of sub-TADs containing ISG gene clusters, thereby promoting the coordinated expression of ISGs.

### Regulation of immune gene activation by specific chromatin loops in response to *Brucella* infection

To better understand how *Brucella* infection impacts gene regulation, we examined the role of chromatin loops, which are crucial for enhancer-promoter interactions, in immune gene activation.

**Figure 5:**
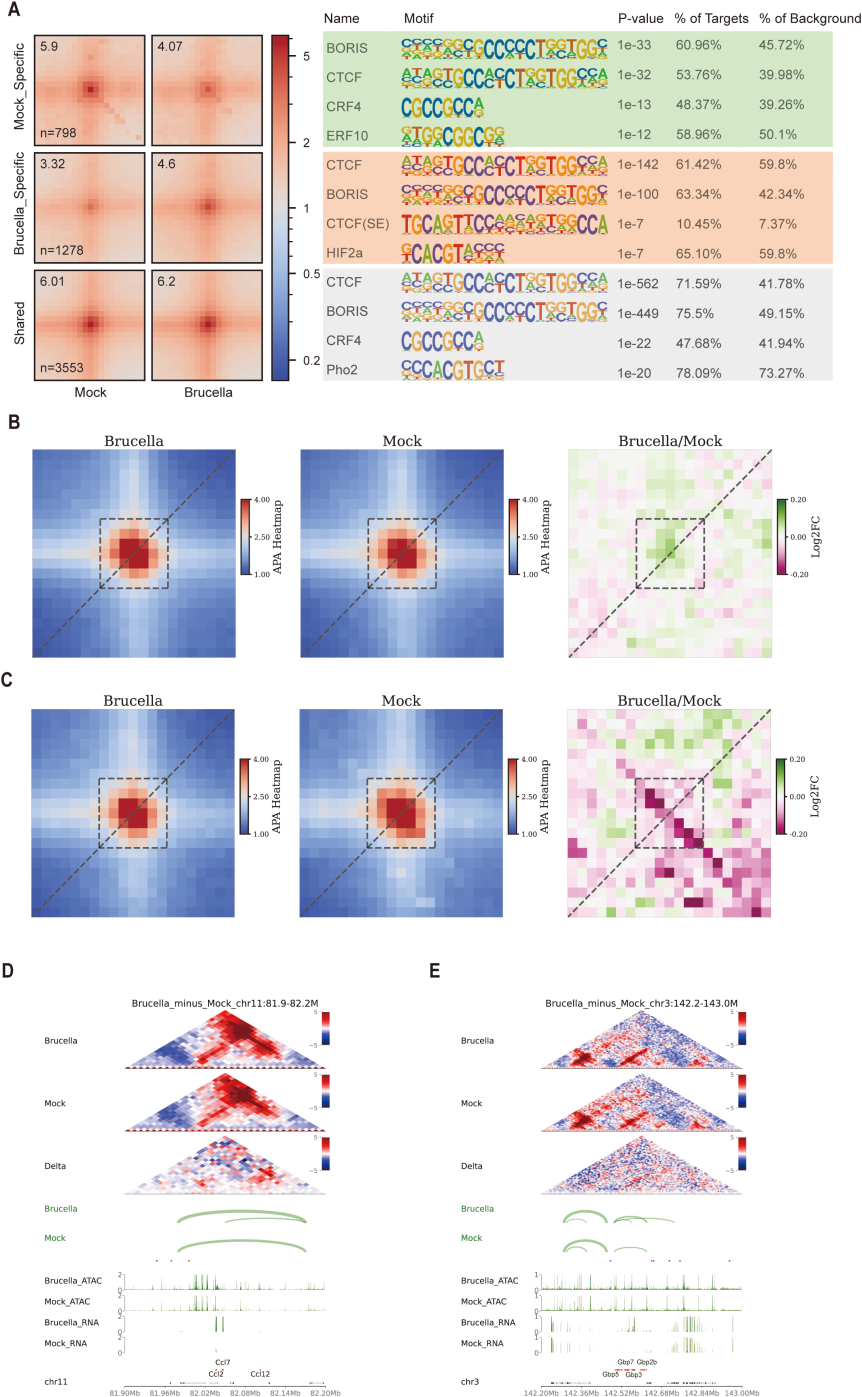
*Brucella* infection modulates chromatin looping interactions. **(A)** Aggregate Peak Analysis (APA) depicting the enrichment peaks and motif annotations of specific and conserved chromatin loops before and after Brucella infection. The figure displays the top four results, highlighting the most significant changes in loop interactions and associated motifs. **(B)** APA illustrating the enrichment peaks and differences of loops associated with upregulated genes following Brucella infection. Enhanced loop anchor interactions are indicated in green, reflecting increased chromatin interaction strength. **(C)** APA illustrating the enrichment peaks and differences of loops associated with downregulated genes following Brucella infection. Weakened loop anchor interactions are indicated in red, reflecting decreased chromatin interaction strength. **(D)** Multi-omics data visualization for the chr11:81.9-82.2M region. In the interaction heatmap, the signals for both *Brucella*-infected and Mock samples were normalized by Z-score, while the Delta heatmap represents the differential interactions between Brucella and Mock groups (red: stronger interactions in Brucella; blue: stronger interactions in Mock). The Loop track displays the identified chromatin loops, with the connecting line width indicating the loop’s q-value (statistical significance). The triangular arrows indicate the orientation of CTCF motifs, with red arrows representing the forward (+) direction and blue arrows denoting the reverse (-) direction. The ATAC track and RNA track show chromatin accessibility (ATAC-seq signal) and transcriptional activity (RNA-seq signal) in the region, respectively, while the Gene track annotates the genomic positions of genes. **(E)** Multi-omics data visualization for the chr3:142.2-143M region. T In the interaction heatmap, the signals for both *Brucella*-infected and Mock samples were normalized by Z-score, while the Delta heatmap represents the differential interactions between Brucella and Mock groups (red: stronger interactions in Brucella; blue: stronger interactions in Mock). The Loop track displays the identified chromatin loops, with the connecting line width indicating the loop’s q-value (statistical significance). The triangular arrows indicate the orientation of CTCF motifs, with red arrows representing the forward (+) direction and blue arrows denoting the reverse (-) direction. The ATAC track and RNA track show chromatin accessibility (ATAC-seq signal) and transcriptional activity (RNA-seq signal) in the region, respectively, while the Gene track annotates the genomic positions of genes.

Utilizing the Mustache software, we identified 10,556 loops in the *Brucella*-infected group and 9,700 loops in the mock-treated group. Additionally, we detected 1,278 loops that were enhanced and 798 loops that were weakened following infection. Aggregation peak analysis (APA) revealed significant changes in both enhanced and weakened loops. Motif analysis of regions with differential loops, including enhanced, weakened, and stable loops, showed enrichment of the structural protein CTCF and its paralogue BORIS(CTCFL) across all three loop categories (**Figure 5A**). These findings suggest that CTCF plays a crucial role in regulating the formation and disassembly of host chromatin loops before and after *Brucella* infection.

Motifs from differential ATAC peaks can be used to identify transcription factors with biological functions. Using the Diffbind software, we identified 18,643 *Brucella* infection-specific peaks and 27,013 mock-specific peaks (**Supplementary Figure 5A**). Functional annotation of these differential peaks revealed an increase in distal intergenic peaks in the infected group (45.27% vs. 32.15%) (**Supplementary Figures 5B, C**). Conversely, the proportion of peaks within genes and promoters was significantly reduced. We reclassified these differential peaks into promoter, gene body, and distal regions and performed motif analysis using the Homer software. Interestingly, we found significant enrichment of CTCF in all three regions (promoter, gene body, and distal) in *Brucella* infection-specific peaks (**Supplementary Figure 5E**). This indicates that following *Brucella* infection, CTCF binds to more regulatory regions. To verify this, we used the FIMO software (39) to identify CTCF binding sites in the mouse genome and calculated the proportion of these sites in specific ATAC peaks. We found that the proportion of specific peaks in the *Brucella*-infected group was significantly higher than in the mock group (**Supplementary Figure 5D**). Further analysis of chromatin accessibility signals at these predicted CTCF sites showed significantly higher signals in the infected group compared to the mock group (**Supplementary Figure 5F**). Footprint analysis of ATAC peaks also revealed slight increases in signals for CTCF and CTCFL transcription factors following infection (**Supplementary Figures 5G, H**). Collectively, these findings reveal a correlation between *Brucella* infection and altered CTCF binding patterns.

By screening all differential gene loci for loop changes and performing aggregation peak analysis, we found that loop signals were stronger for upregulated genes (**Figure 5B**) and weaker for downregulated genes (**Figure 5C**), indicating that loop strength at gene loci affects gene expression. Further analysis of ISG gene locus loops revealed significant activation of *Ccl2*, *Ccl7*, and *Ccl12* following *Brucella* infection. In the differential heatmap, we detected a clear hotspot of enhanced interactions downstream of these gene loci and a novel loop formation (**Figure 5D**). Similarly, we observed enhanced interactions and the formation of two new loop structures near the *Gbp3*/*Gbp5*/*Gbp7*/*Gbp2b* gene locus (**Figure 5E**). To validate the stability of these chromatin loops across biological replicates, we employed virtual 4C analysis to assess interaction changes at one end of the novel chromatin loops. The results demonstrated that the interaction strength of the novel loops was consistently higher in *Brucella*-infected groups compared to mock controls across biological replicates (**Supplementary Figures 5I, J**).

In summary, our observations are consistent with a model where *Brucella* infection correlates with alterations in host chromatin looping patterns, which may influence transcriptional regulation. These topological changes coincide with immune gene activation, suggesting potential involvement of 3D genome organization in host-pathogen interactions.

## Discussion

**Figure 6:**
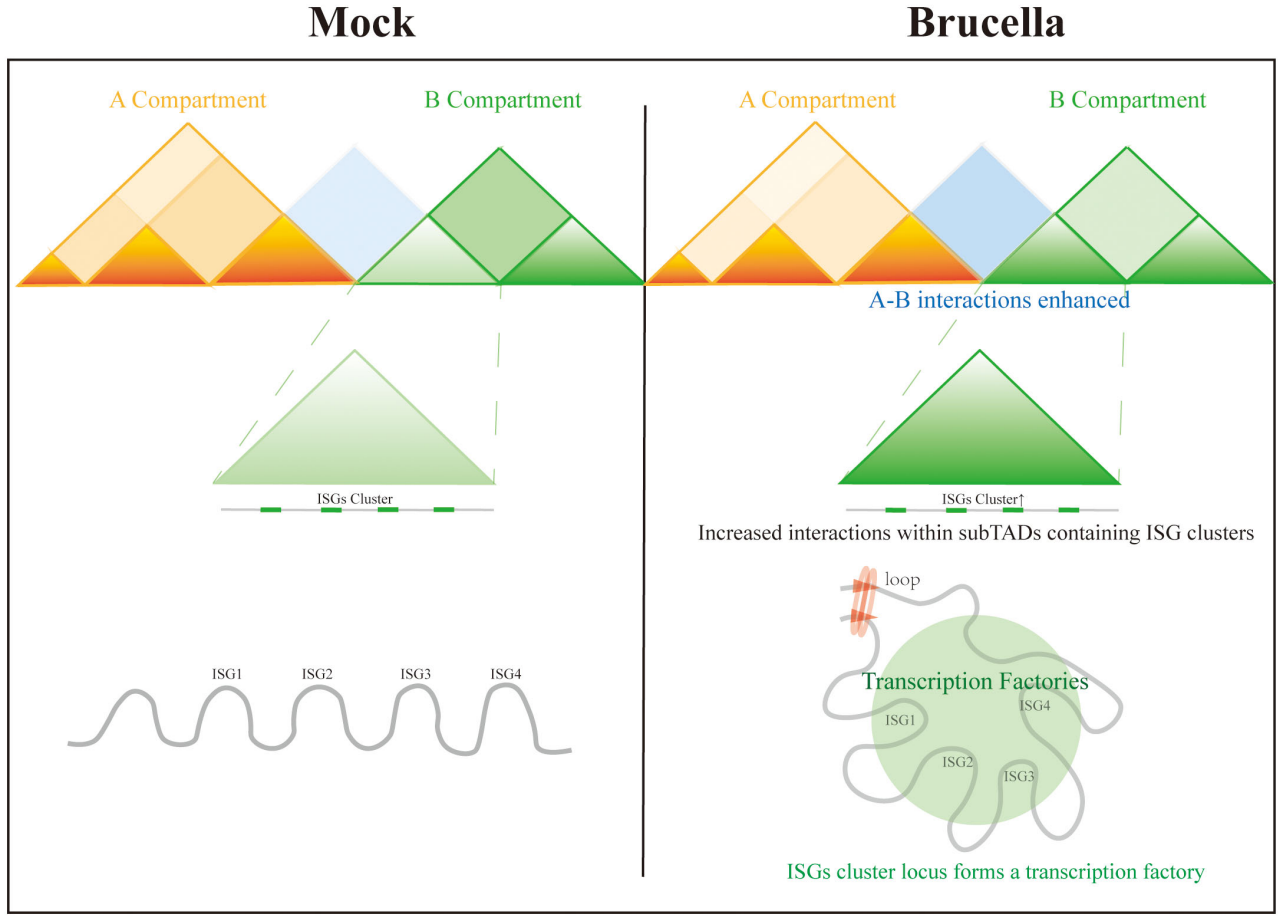
*Brucella* infection induces chromatin restructuring in host cells to activate immune responses.

In this study, we demonstrate that *Brucella* infection induces a global reorganization of host chromatin architecture, marked by enhanced short-range chromosomal interactions and diminished long-range compartmentalization. Specifically, interactions within B-B homotypic compartments were attenuated, while interactions between A and B heterotypic mixing compartments were enhanced. Parallel epigenomic profiling revealed increased chromatin accessibility at promoter regions, correlating with transcriptional activation of immune-related loci. Notably, we identified that the coordinated upregulation of ISGs was mechanistically linked to strengthened intra-sub-TAD interactions within ISG-enriched genomic clusters. These structural rearrangements facilitated the formation of transcriptionally active hubs, while novel chromatin loop formations may further orchestrate the synchronized expression of ISG clusters—a phenomenon potentially critical for rapid immune activation (**Figure 6**).

We hypothesize that host cells may facilitate bacterial clearance through chromatin remodeling via the following three mechanisms:

1. Rapid activation of host defense genes. For instance, we observed significant upregulation of *Tnf* and *Gbp* genes, which may directly enhance antibacterial effects by disrupting bacterial membrane integrity (40) or activating inflammasomes (41).
2. Induction of chemokines to recruit immune cells. Host cells upregulate *Ccl* gene family members (e.g., *Ccl2*), which bind to receptors (e.g., CCR2) on immune cells, recruiting monocytes, macrophages, and memory T cells to infection sites. For example, in animal models, *Ccl2* overexpression markedly enhances macrophage-mediated pathogen phagocytosis (42).
3. Suppression of host cell proliferation to restrict bacterial replication. By modulating cell cycle regulators (e.g., Ccna2/Ccnb2), host cells may actively arrest proliferation, thereby limiting intracellular resources for bacterial survival.

The host immune response to microbial invasion requires rapid, coordinated activation of diverse antimicrobial effector genes. While interferons (especially IFN-γ) are known to orchestrate antibacterial immunity through induction of hundreds of interferon-stimulated genes encoding antimicrobial proteins (4), the mechanisms governing their spatiotemporal coordination remain poorly understood. Evolutionary adaptation has led to the spatial clustering of functionally related immune genes within shared chromatin domains (e.g., sub-TADs), facilitating their coregulation. Our studies demonstrate significant enrichment of immune genes within cooperative sub-TADs, suggesting this genomic architecture enhances rapid transcriptional responses to infection.

Notably, we observed that Brucella infection strengthens intra-cluster chromatin interactions, potentially enabling efficient gene coregulation through shared enhancer elements. Most strikingly, infection induces *de novo* chromatin loop formation at ISG loci, with these dynamic structural changes correlating with synchronized ISG activation. This spatial reorganization creates specialized “transcriptional factories” that optimize rapid ISG induction following interferon signaling. Supporting this model, studies in M. tuberculosis-infected macrophages reveal that liquid-liquid phase separation (LLPS) at GBP clusters promotes coordinated GBP family expression (18). Together, these findings establish that dynamic chromatin remodeling - including subTAD reorganization, loop formation, and LLPS - represents an evolutionarily conserved mechanism for orchestrating antimicrobial gene expression programs during infection.

Of particular interest is the *Gbp* gene family, interferon-inducible GTPases with dual roles in direct pathogen clearance and inflammasome regulation (43–46). Notably, GBP proteins exhibit cytotoxicity; their expression can cause Golgi fragmentation and cell death (47). This suggests that host cells must have precise mechanisms to regulate the expression of this gene family. We revealed that murine *Gbp* clusters are strategically positioned near A/B compartment boundaries in a repressed state under homeostasis. Following infection, one locus underwent compartment switching (B→A), while the other established new contacts with adjacent A Compartment, enabling precise transcriptional activation (**Figure 3F**, **Supplementary Figure 3F**). This spatial regulation may balance the cytotoxic consequences of *Gbp* overexpression (e.g., Golgi destabilization) with the necessity for pathogen control—a paradigm of evolutionary optimization for immunity-related gene clusters.

Epigenetic modifications exert long-lasting and persistent effects on gene expression and phenotypes (48–50). Although direct evidence is currently lacking to demonstrate that *Brucella*-induced chromatin remodeling is heritable across cell generations, our observations suggest that infection may profoundly reprogram cellular functionality. Notably, studies reveal that Brucella infection drives macrophage polarization, shifting from classically activated (M1) to alternatively activated (M2) phenotypes (51). Supporting this, single-cell transcriptomic data from human brucellosis patients further indicate that acute infection promotes monocyte differentiation into myeloid-derived suppressor cell (MDSC) subsets—a phenomenon potentially linked to persistent immunomodulation (52). Given that a subset of brucellosis patients transition from acute infection to chronic disease, understanding the impact of *Brucella* infection on host chromatin structure will not only provide novel strategies for combating *Brucella* infection during the acute phase but also offer fresh insights into the mechanisms by which brucellosis promotes its chronic progression.

## Data Availability

The raw sequencing data of Hi-C, ATAC-seq, and RNA-seq generated in this study have been deposited in the Genome Sequence Archive in National Genomics Data Center (33), China National Center for Bioinformation/Beijing Institute of Genomics, Chinese Academy of Sciences (GSA: CRA022765) that are publicly accessible at https://ngdc.cncb.ac.cn/gsa (34). The analyzed data and code that support the findings of this study are available from the corresponding author upon reasonable request.

## Supplementary material

Supplementary Figure 1
*Brucella* Infection Alters Host Cell Phenotype and Chromatin Organization. **(A)** Morphological appearance of control group cells after 48 hours of culture. Cells form clusters, indicating typical growth patterns under baseline conditions. **(B)** Morphological appearance of cells infected with Brucella after 48 hours of culture. Cells exhibit a sparser distribution, with some cells displaying pseudopodia, indicative of potential cellular stress or response to infection. **(C)** HiCRep statistics presenting the correlation heatmap of Hi-C libraries before and after *Brucella* infection. The heatmap illustrates the reproducibility and consistency of chromatin interaction data across samples. **(D)** Statistics of the chromatin attenuation index for A/B compartments. The index provides a quantitative measure of changes in chromatin density and organization between the two compartments. **(E)** Chromatin interaction heatmap of chromosome 2 in host cells after *Brucella* infection. The heatmap visualizes the frequency and strength of chromatin interactions within this genomic region. **(F)** Chromatin interaction heatmap of chromosome 2 in control group cells. The heatmap provides a baseline representation of chromatin interactions within this genomic region under normal conditions. **(G)** Z-score difference heatmap of chromosome 2 interactions between *Brucella*-infected host cells and control cells. The results highlight regions where interactions are significantly enhanced in A compartments following infection, indicating dynamic changes in chromatin organization. **(H)** Chromatin interaction map of *Brucella*. The *Brucella* genome comprises two chromosomes. Heatmap colors indicate interaction intensity. **(I)** Chromatin accessibility heatmap of gene body regions across five biological replicates in *Brucella*-infected samples.

Supplementary Figure 2 Quality Control of Transcriptome Data, Functional Enrichment of Differentially Expressed Genes, and Display of Selected Gene Loci. **(A)** Heatmap depicting the correlation of gene expression between Brucella-infected and mock-treated groups. Spearman`s rank correlation coefficient was utilized to assess the correlation of gene expression among biological replicates, providing a measure of reproducibility and consistency across samples. **(B)** Principal component analysis of gene expression in samples from infected and control groups. The PCA plot illustrates the variance explained by principal components, highlighting the separation between infected and mock samples based on transcriptional profiles. **(C)** KEGG pathway analysis of differentially expressed genes. The analysis identifies significantly enriched pathways, providing insights into the biological processes and signaling pathways affected by *Brucella* infection. **(D)** Bar chart illustrating the distribution of fold changes for genes in the JAK-STAT signaling pathway. The chart highlights the magnitude of expression changes in key genes involved in this pathway, reflecting the impact of *Brucella* infection on immune signaling. **(E)** GSEA enrichment plot revealing significant enrichment in the activation of the innate immune response. The plot highlights the overrepresentation of genes associated with the innate immune response in the context of *Brucella* infection. **(F)** GSEA enrichment plot revealing significant enrichment in cell chemotaxis. The plot highlights the overrepresentation of genes associated with cell migration and recruitment in response to *Brucella* infection. **(G)** GSEA enrichment plot revealing significant enrichment in response to interferon-beta. The plot highlights the overrepresentation of genes associated with the interferon-beta signaling pathway, indicating a robust antiviral and immune response. **(H)** GSEA enrichment plot revealing significant enrichment in response to interferon-gamma. The plot highlights the overrepresentation of genes associated with the interferon-gamma signaling pathway, indicating a robust immune response to *Brucella* infection. **(I)** ATAC-seq signal and RNA-seq signal around the *Ccl3* gene locus. The red dashed region indicates enhanced chromatin accessibility at this locus in the *Brucella*-infected group. **(J)** ATAC-seq signal and RNA-seq signal around the *Ccl4* gene locus. The red dashed region indicates enhanced chromatin accessibility at this locus in the *Brucella*-infected group. **(K)** ATAC-seq signal and RNA-seq signal around the *Ccl5* gene locus. The red dashed region indicates enhanced chromatin accessibility at this locus in the *Brucella*-infected group. **(L)** ATAC-seq signal and RNA-seq signal around the *Apoe* gene locus. The red dashed region indicates reduced chromatin accessibility at this locus in the *Brucella*-infected group. **(M)** ATAC-seq signal and RNA-seq signal around the *Lyz1* gene locus. The red dashed region indicates reduced chromatin accessibility at this locus in the *Brucella*-infected group. **(N)** ATAC-seq signal and RNA-seq signal around the *Lyz2* gene locus. The red dashed region indicates reduced chromatin accessibility at this locus in the *Brucella*-infected group.

Supplementary Figure 3 Brucella Infection Reshapes Chromatin Compartment Structure. **(A)** APA depicting the interaction strength within A compartment regions, with one adjacent compartment region extended on each side. This analysis provides insights into the changes in chromatin interaction dynamics within A compartments upon Brucella infection. **(B)** APA depicting the interaction strength within B compartment regions, with one adjacent compartment region extended on each side. This analysis provides insights into the changes in chromatin interaction dynamics within B compartments upon *Brucella* infection. **(C)** Bar chart illustrating GO annotations of upregulated genes in stable A compartment regions. The chart highlights the functional categories enriched among genes that remain in the A compartment and are upregulated following infection. **(D)** Bar chart illustrating GO annotations of upregulated genes in stable B compartment regions. The chart highlights the functional categories enriched among genes that remain in the B compartment and are upregulated following infection. **(E)** Dynamic changes in the *Ifi214/Ifi209/Ifi208/Ifi207* gene locus region before and after Brucella infection. The compartment vector indicates a transition of this gene locus from the B compartment to the A compartment. In the figure, the blue area represents the B compartment, and the red area represents the A compartment. This transition reflects significant changes in chromatin organization associated with gene activation. **(F)** Dynamic changes in the *Gbp3/Gbp5/Gbp7* gene locus region before and after Brucella infection. The compartment vector indicates a transition of this gene locus from the B compartment to the A compartment. In the figure, the blue area represents the B compartment, and the red area represents the A compartment. This transition reflects significant changes in chromatin organization associated with gene activation.

Supplementary Figure 4 The Impact of Sub-TADs Internal Interaction Strength on Gene Expression. **(A)** Aggregate Domain Analysis (ADA) depicting the interaction strength within sub-TADs of different expression levels, with one TAD extended on each side. The analysis provides insights into how chromatin interactions within sub-TADs correlate with gene expression levels. **(B)** Visualization of the average insulation index of sub-TADs regions with different expression levels. The insulation index reflects the boundary strength of sub-TADs and its potential impact on gene regulation. **(C)** ADA showing the interactions within the sub-TADs of Upgene Polymorphic gene loci and their differences between the infection and Mock groups. The analysis highlights changes in chromatin interaction patterns associated with upregulated polymorphic genes. **(D)** ADA showing the interactions within the sub-TADs of Downgene Polymorphic gene loci and their differences between the infection and Mock groups. The analysis highlights changes in chromatin interaction patterns associated with downregulated polymorphic genes. **(E)** ADA showing the interactions within the sub-TADs of Upgene Independent gene loci and their differences between the infection and Mock groups. The analysis provides insights into the chromatin interaction dynamics of independently regulated genes that are upregulated upon infection. **(F)** ADA showing the interactions within the sub-TADs of Downgene Independent gene loci and their differences between the infection and Mock groups. The analysis provides insights into the chromatin interaction dynamics of independently regulated genes that are downregulated upon infection. **(G)** Visualization of ATAC signal changes in the Upgene cluster gene locus region. The figure highlights alterations in chromatin accessibility associated with upregulated gene clusters, reflecting changes in regulatory landscapes. **(H)** Visualization of ATAC signal changes in the Downgene cluster gene locus region. The figure highlights alterations in chromatin accessibility associated with downregulated gene clusters, reflecting changes in regulatory landscapes.

Supplementary Figure 5
*Brucella* Infection Induces Specific Chromatin Accessibility Peaks Enriched for CTCF Motif. **(A)** Venn diagram illustrating the overlap and unique differential peaks identified between the Brucella-infected and mock-treated groups. The diagram provides a visual representation of the distinct and shared chromatin accessibility changes induced by *Brucella* infection. **(B)** Pie chart depicting the genomic annotations of ATAC-Seq peaks specific to the mock-treated group. The chart categorizes peaks by their proximity to different genomic elements (e.g., promoters, gene bodies, intergenic regions), highlighting the distribution of accessible chromatin regions under baseline conditions. **(C)** Pie chart depicting the genomic annotations of ATAC-Seq peaks specific to the Brucella-infected group. The chart categorizes peaks by their proximity to different genomic elements, highlighting the distribution of accessible chromatin regions altered by Brucella infection. **(D)** Enrichment analysis of ATAC signals and motif statistics in specific peak regions. The analysis identifies significant enrichment of chromatin accessibility signals and the presence of specific motifs, such as CTCF, within differentially accessible regions. **(E)** Bar chart showing the number of peaks containing the CTCF motif in both the Brucella-infected and mock-treated groups. The chart provides a quantitative comparison of CTCF motif enrichment in accessible chromatin regions between the two conditions. **(F)** Heatmap illustrating the enrichment of chromatin accessibility signals in regions containing the CTCF motif. The heatmap provides a visual representation of the relative accessibility levels across samples, highlighting regions with significant changes in accessibility following *Brucella* infection. **(G)** Visualization of CTCF footprint signals in peak regions. The figure displays the presence and strength of CTCF binding sites within differentially accessible regions, providing insights into the role of CTCF in chromatin remodeling. **(H)** Visualization of CTCF-like (CTCFL) footprint signals in peak regions. The figure displays the presence and strength of CTCFL binding sites within differentially accessible regions, providing insights into the potential involvement of CTCFL in chromatin remodeling. **(I)** Local view of ATAC-seq, RNA-seq, and virtual 4C signals near the Ccl2/Ccl7/Ccl12 gene loci. The virtual 4C signal uses a bait region (highlighted in light green) at one end of the loop (chr11:82.17-82.18 Mb) to detect interaction signals between this region and other genomic loci. **(J)** Local view of ATAC-seq, RNA-seq, and virtual 4C signals near the *Gbp3*/*Gbp5*/*Gbp7*/*Gbp2b* gene loci. The virtual 4C signal uses a bait region (highlighted in light green) at one end of the loop (chr3:142.49-142.5 Mb) to detect interaction signals between this region and other genomic loci.

Supplementary Table 1 Hi-C data quality control results. Sheet1 contains the alignment quality control for Hi-C data. Sheet2 includes the PCR duplicate analysis for the Hi-C library. Sheet3 presents the cis/trans results for the Hi-C library.
