# Supplementary figures and images for: Brucella infection induces chromatin restructuring in host cells to activate immune responses
Source: Front Immunol. 2025 Jun 5;16:1574006. doi: 10.3389/fimmu.2025.1574006 (PMC12176892; doi:10.3389/fimmu.2025.1574006)

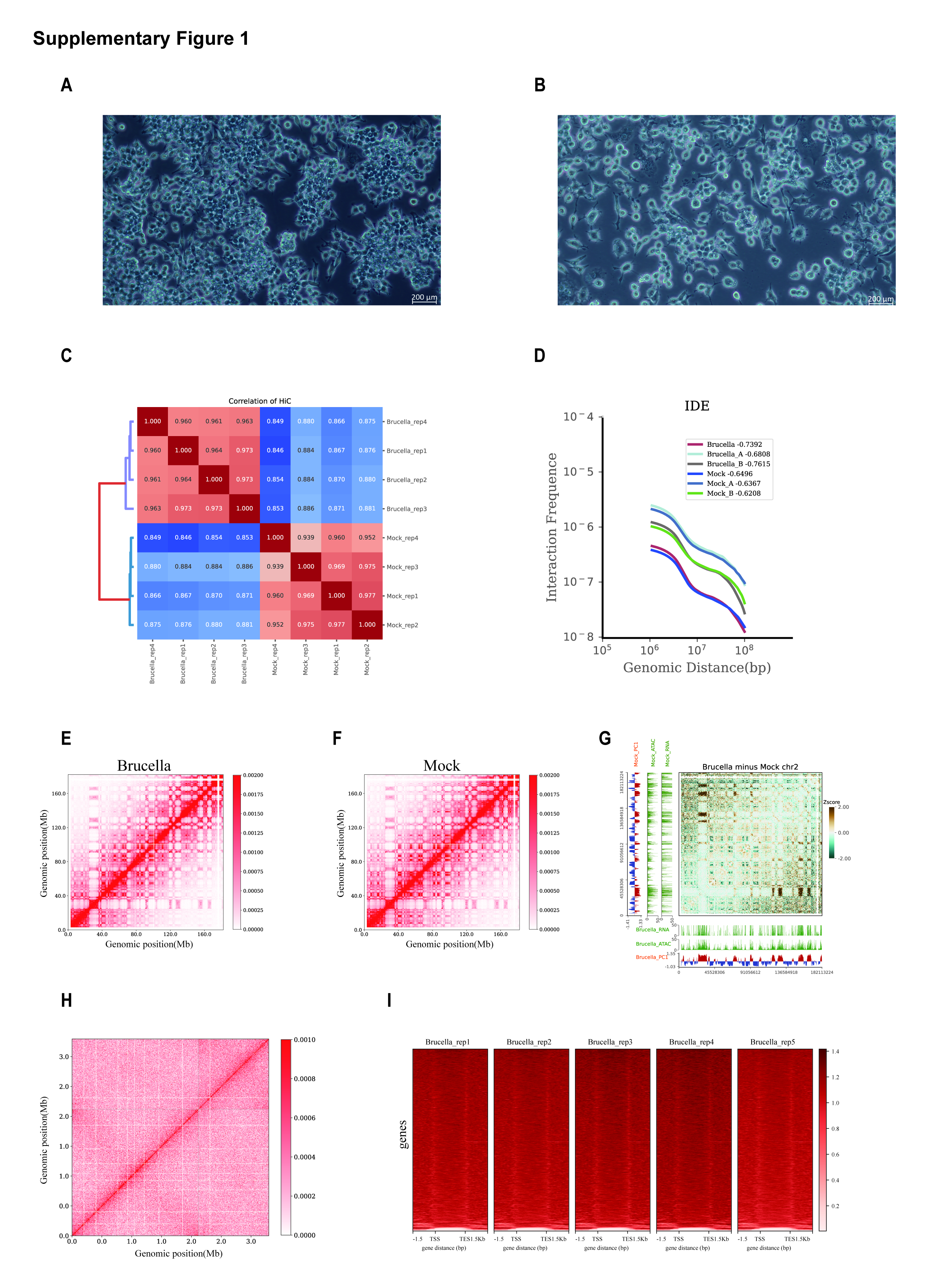

Supplement: Supplementary Figure 1 — Brucella Infection Alters Host Cell Phenotype and Chromatin Organization. (A) Morphological appearance of control group cells after 48 hours of culture. Cells form clusters, indicating typical growth patterns under baseline conditions. (B) Morphological appearance of cells infected with Brucella after 48 hours of culture. Cells exhibit a sparser distribution, with some cells displaying pseudopodia, indicative of potential cellular stress or response to infection. (C) HiCRep statistics presenting the correlation heatmap of Hi-C libraries before and after Brucella infection. The heatmap illustrates the reproducibility and consistency of chromatin interaction data across samples. (D) Statistics of the chromatin attenuation index for A/B compartments. The index provides a quantitative measure of changes in chromatin density and organization between the two compartments. (E) Chromatin interaction heatmap of chromosome 2 in host cells after Brucella infection. The heatmap visualizes the frequency and strength of chromatin interactions within this genomic region. (F) Chromatin interaction heatmap of chromosome 2 in control group cells. The heatmap provides a baseline representation of chromatin interactions within this genomic region under normal conditions. (G) Z-score difference heatmap of chromosome 2 interactions between Brucella-infected host cells and control cells. The results highlight regions where interactions are significantly enhanced in A compartments following infection, indicating dynamic changes in chromatin organization. (H) Chromatin interaction map of Brucella. The Brucella genome comprises two chromosomes. Heatmap colors indicate interaction intensity. (I) Chromatin accessibility heatmap of gene body regions across five biological replicates in Brucella-infected samples. [file Image1.tif]

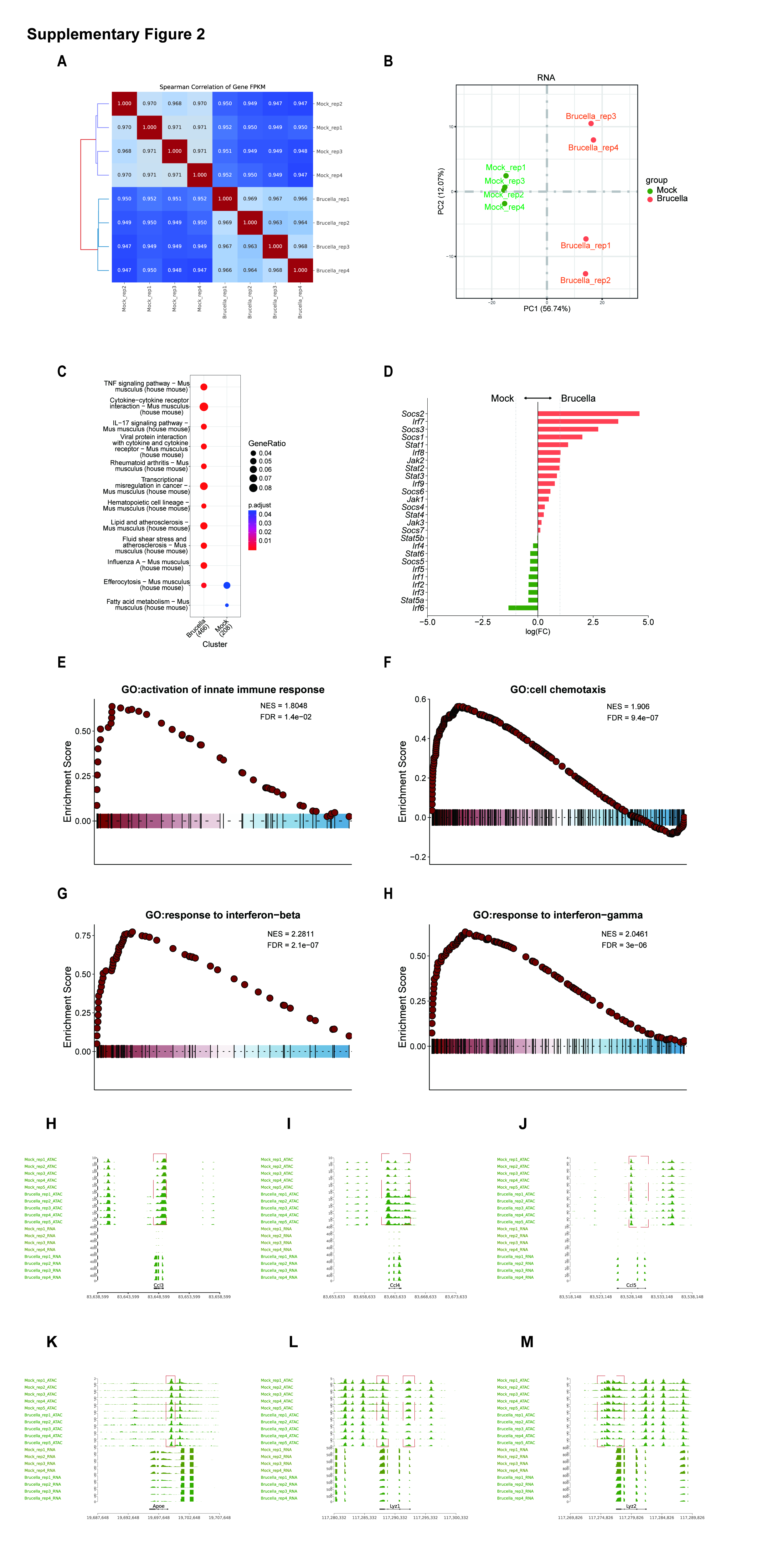

Supplement: Supplementary Figure 2 — Quality Control of Transcriptome Data, Functional Enrichment of Differentially Expressed Genes, and Display of Selected Gene Loci. (A) Heatmap depicting the correlation of gene expression between Brucella-infected and mock-treated groups. Spearman`s rank correlation coefficient was utilized to assess the correlation of gene expression among biological replicates, providing a measure of reproducibility and consistency across samples. (B) Principal component analysis of gene expression in samples from infected and control groups. The PCA plot illustrates the variance explained by principal components, highlighting the separation between infected and mock samples based on transcriptional profiles. (C) KEGG pathway analysis of differentially expressed genes. The analysis identifies significantly enriched pathways, providing insights into the biological processes and signaling pathways affected by Brucella infection. (D) Bar chart illustrating the distribution of fold changes for genes in the JAK-STAT signaling pathway. The chart highlights the magnitude of expression changes in key genes involved in this pathway, reflecting the impact of Brucella infection on immune signaling. (E) GSEA enrichment plot revealing significant enrichment in the activation of the innate immune response. The plot highlights the overrepresentation of genes associated with the innate immune response in the context of Brucella infection. (F) GSEA enrichment plot revealing significant enrichment in cell chemotaxis. The plot highlights the overrepresentation of genes associated with cell migration and recruitment in response to Brucella infection. (G) GSEA enrichment plot revealing significant enrichment in response to interferon-beta. The plot highlights the overrepresentation of genes associated with the interferon-beta signaling pathway, indicating a robust antiviral and immune response. (H) GSEA enrichment plot revealing significant enrichment in response to interferon-gamma. The plot highligh [file Image2.tif]

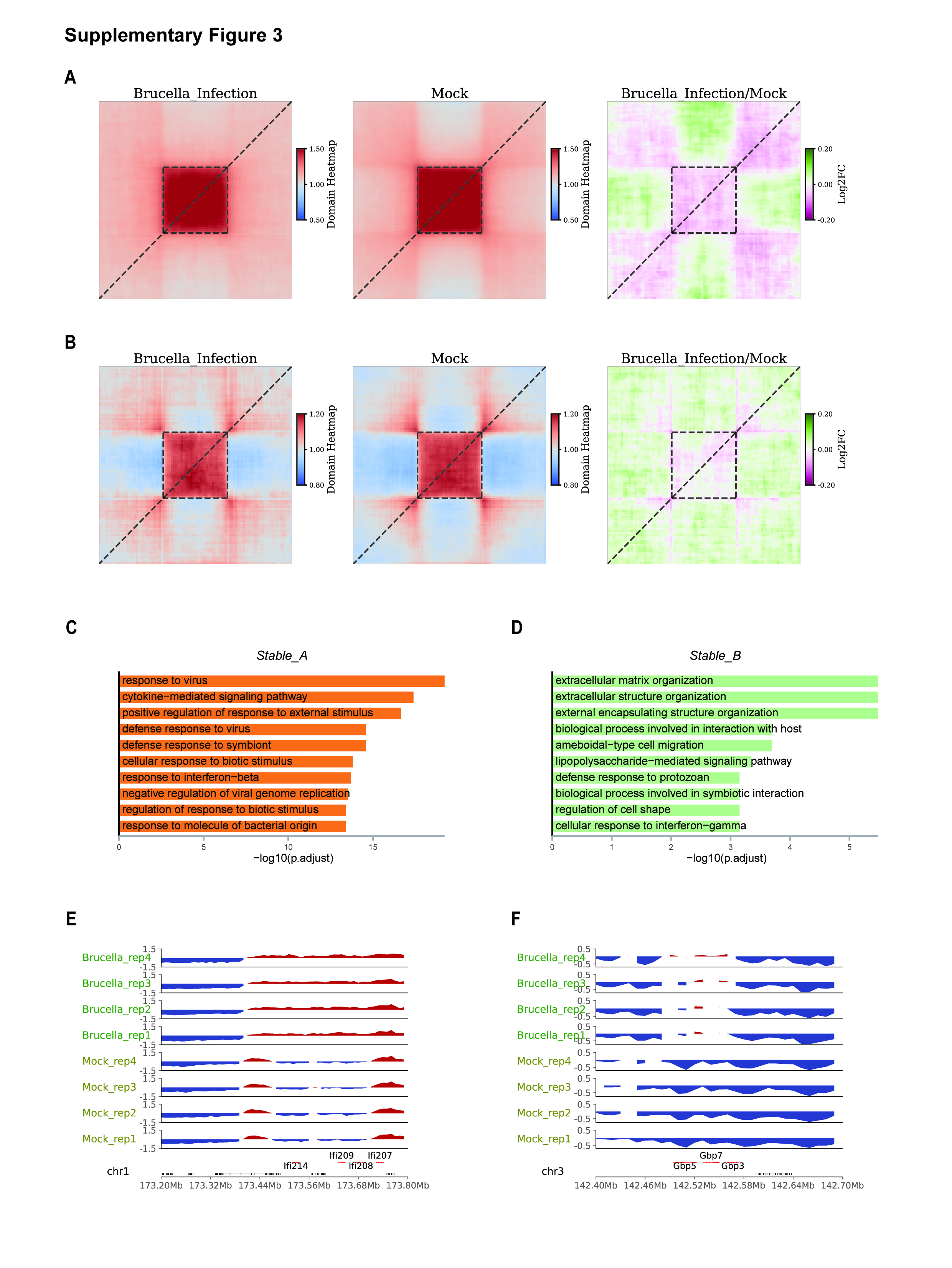

Supplement: Supplementary Figure 3 — Brucella Infection Reshapes Chromatin Compartment Structure. (A) APA depicting the interaction strength within A compartment regions, with one adjacent compartment region extended on each side. This analysis provides insights into the changes in chromatin interaction dynamics within A compartments upon Brucella infection. (B) APA depicting the interaction strength within B compartment regions, with one adjacent compartment region extended on each side. This analysis provides insights into the changes in chromatin interaction dynamics within B compartments upon Brucella infection. (C) Bar chart illustrating GO annotations of upregulated genes in stable A compartment regions. The chart highlights the functional categories enriched among genes that remain in the A compartment and are upregulated following infection. (D) Bar chart illustrating GO annotations of upregulated genes in stable B compartment regions. The chart highlights the functional categories enriched among genes that remain in the B compartment and are upregulated following infection. (E) Dynamic changes in the Ifi214/Ifi209/Ifi208/Ifi207 gene locus region before and after Brucella infection. The compartment vector indicates a transition of this gene locus from the B compartment to the A compartment. In the figure, the blue area represents the B compartment, and the red area represents the A compartment. This transition reflects significant changes in chromatin organization associated with gene activation. (F) Dynamic changes in the Gbp3/Gbp5/Gbp7 gene locus region before and after Brucella infection. The compartment vector indicates a transition of this gene locus from the B compartment to the A compartment. In the figure, the blue area represents the B compartment, and the red area represents the A compartment. This transition reflects significant changes in chromatin organization associated with gene activation. [file Image3.tif]

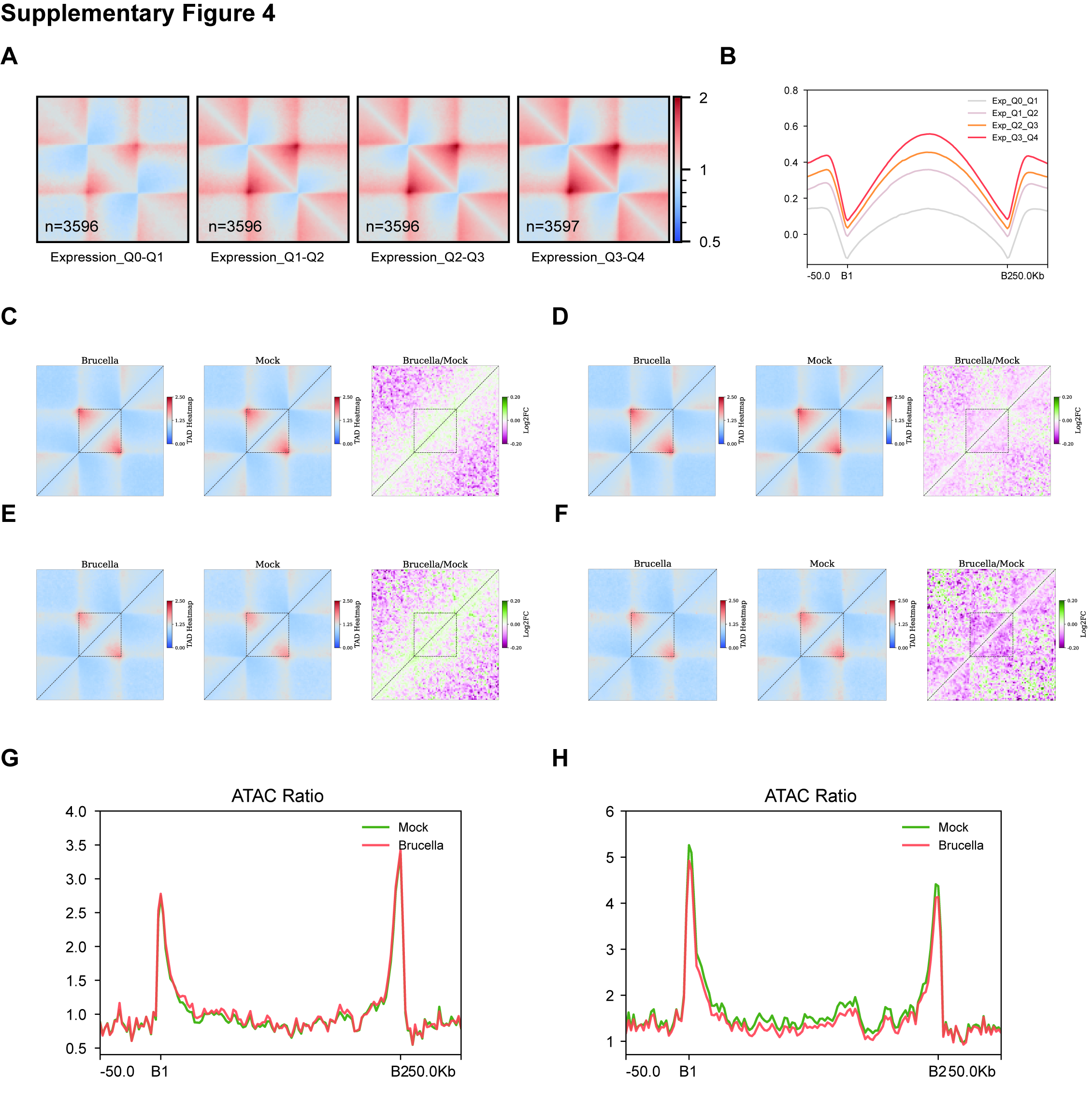

Supplement: Supplementary Figure 4 — The Impact of Sub-TADs Internal Interaction Strength on Gene Expression. (A) Aggregate Domain Analysis (ADA) depicting the interaction strength within sub-TADs of different expression levels, with one TAD extended on each side. The analysis provides insights into how chromatin interactions within sub-TADs correlate with gene expression levels. (B) Visualization of the average insulation index of sub-TADs regions with different expression levels. The insulation index reflects the boundary strength of sub-TADs and its potential impact on gene regulation. (C) ADA showing the interactions within the sub-TADs of Upgene Polymorphic gene loci and their differences between the infection and Mock groups. The analysis highlights changes in chromatin interaction patterns associated with upregulated polymorphic genes. (D) ADA showing the interactions within the sub-TADs of Downgene Polymorphic gene loci and their differences between the infection and Mock groups. The analysis highlights changes in chromatin interaction patterns associated with downregulated polymorphic genes. (E) ADA showing the interactions within the sub-TADs of Upgene Independent gene loci and their differences between the infection and Mock groups. The analysis provides insights into the chromatin interaction dynamics of independently regulated genes that are upregulated upon infection. (F) ADA showing the interactions within the sub-TADs of Downgene Independent gene loci and their differences between the infection and Mock groups. The analysis provides insights into the chromatin interaction dynamics of independently regulated genes that are downregulated upon infection. (G) Visualization of ATAC signal changes in the Upgene cluster gene locus region. The figure highlights alterations in chromatin accessibility associated with upregulated gene clusters, reflecting changes in regulatory landscapes. (H) Visualization of ATAC signal changes in the Downgene cluster gene locus region. The figure highlights alte [file Image4.tif]

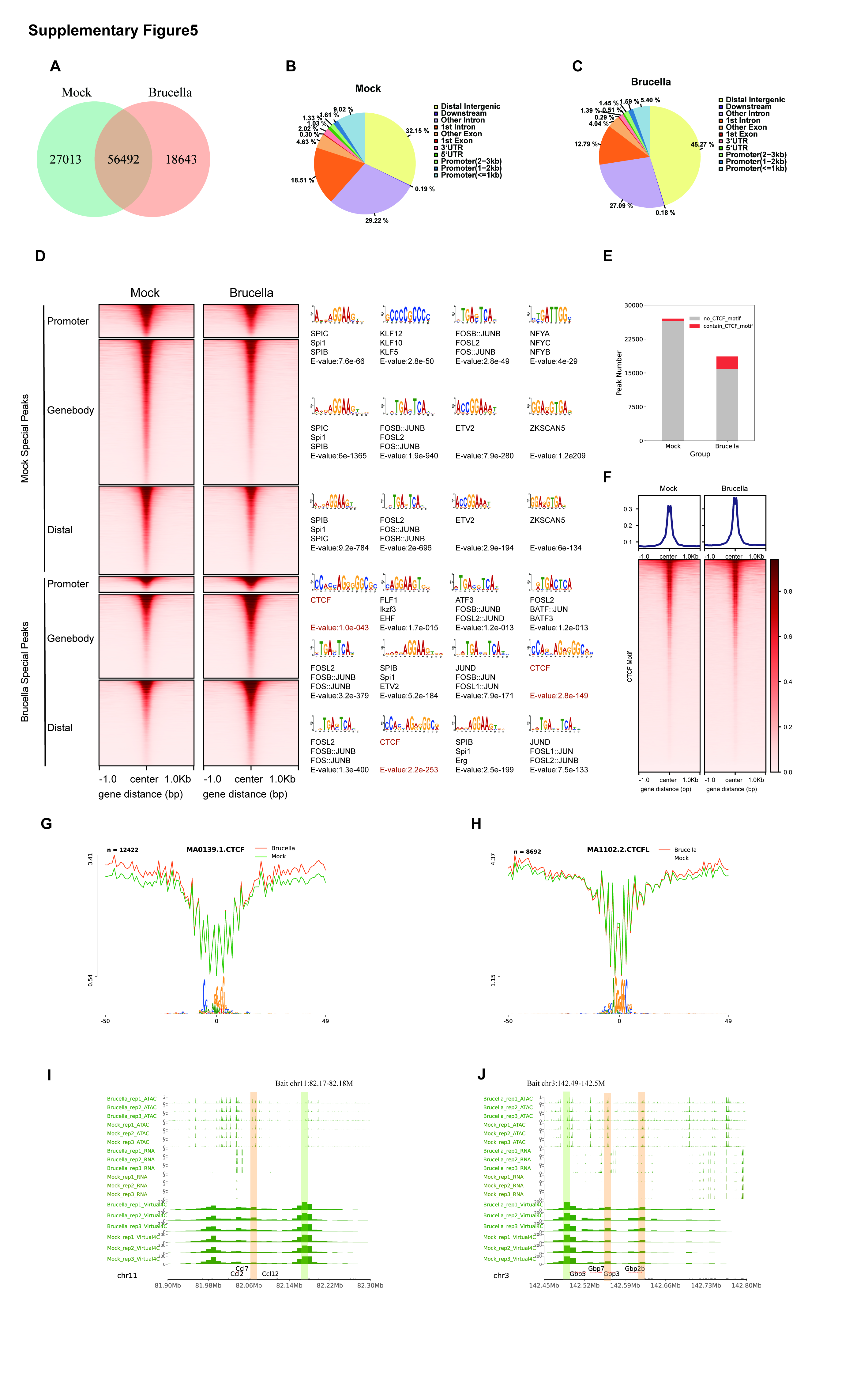

Supplement: Supplementary Figure 5 — Brucella Infection Induces Specific Chromatin Accessibility Peaks Enriched for CTCF Motif. (A) Venn diagram illustrating the overlap and unique differential peaks identified between the Brucella-infected and mock-treated groups. The diagram provides a visual representation of the distinct and shared chromatin accessibility changes induced by Brucella infection. (B) Pie chart depicting the genomic annotations of ATAC-Seq peaks specific to the mock-treated group. The chart categorizes peaks by their proximity to different genomic elements (e.g., promoters, gene bodies, intergenic regions), highlighting the distribution of accessible chromatin regions under baseline conditions. (C) Pie chart depicting the genomic annotations of ATAC-Seq peaks specific to the Brucella-infected group. The chart categorizes peaks by their proximity to different genomic elements, highlighting the distribution of accessible chromatin regions altered by Brucella infection. (D) Enrichment analysis of ATAC signals and motif statistics in specific peak regions. The analysis identifies significant enrichment of chromatin accessibility signals and the presence of specific motifs, such as CTCF, within differentially accessible regions. (E) Bar chart showing the number of peaks containing the CTCF motif in both the Brucella-infected and mock-treated groups. The chart provides a quantitative comparison of CTCF motif enrichment in accessible chromatin regions between the two conditions. (F) Heatmap illustrating the enrichment of chromatin accessibility signals in regions containing the CTCF motif. The heatmap provides a visual representation of the relative accessibility levels across samples, highlighting regions with significant changes in accessibility following Brucella infection. (G) Visualization of CTCF footprint signals in peak regions. The figure displays the presence and strength of CTCF binding sites within differentially accessible regions, providing insights into the role of CTCF in chro [file Image5.tif]
